# Myeloid-derived suppressor cell inhibits T-cell-based defense against *Klebsiella pneumoniae* infection via IDO1 production

**DOI:** 10.1371/journal.ppat.1012979

**Published:** 2025-03-17

**Authors:** Qi Xu, Xiaoxuan Liu, Heng Heng, Han Wang, Kaichao Chen, Edward Wai-Chi Chan, Guan Yang, Sheng Chen

**Affiliations:** 1 State Key Laboratory of Chemical Biology and Drug Discovery and the Department of Food Science and Nutrition, The Hong Kong Polytechnic University, Kowloon, Hong Kong SAR; 2 Department of Infectious Diseases and Public Health, Jockey Club College of Veterinary Medicine and Life Sciences, City University of Hong Kong, Kowloon, Hong Kong SAR; 3 Shenzhen Key Laboratory for Food Biological Safety Control, Food Safety and Technology Research Centre, The Hong Kong PolyU Shenzhen Research Institute, Shenzhen, People’s Republic of China; Virginia Commonwealth University School of Medicine, UNITED STATES OF AMERICA

## Abstract

*Klebsiella pneumoniae* (*Kp*) is responsible for a wide range of infections, including pneumonia, sepsis, and urinary tract infections. However, the treatment options are limited due to the continuous evolution of drug-resistant and hypervirulent variants. It is crucial to investigate the mechanisms behind the high mortality rate of hypervirulent *Kp* (hv*Kp*) strains to develop new strategies for preventing hv*Kp* from evading the host’s defenses and improving treatment effectiveness for these fatal infections. In this study, we used a hv*Kp*-induced mouse bacteremia model and performed single-cell RNA sequencing to investigate the effects of hv*Kp* infection. Our findings demonstrated that hv*Kp* infection led to a decrease in lymphocytes (lymphopenia), attributed to impaired proliferation and apoptosis. The infiltration of myeloid-derived suppressor cells (MDSCs) in the infected lungs was confirmed to suppress T cell proliferation, leading to lymphopenia. We further identified that hv*Kp* promotes tryptophan metabolism in infected lungs, enhancing the immunosuppressive activity of MDSCs by inducing the production of the enzyme IDO1. Our *ex vivo* inhibition experiment revealed that L-kynurenine, a product of tryptophan metabolism, inhibits T-cell proliferation and induces T-cell apoptosis, further suppressing T-cell mediated responses against bacteria. Importantly, when we knocked out the *Ido1* gene or inhibited IDO1 expression using a specific inhibitor 1-MT in mice, we observed a significant enhancement in T-cell mediated responses against hv*Kp*. These findings highlight the crucial role of MDSCs in hv*Kp*-induced bacteremia and suggest a promising immunotherapeutic approach by inhibiting IDO1 production to combat infectious diseases.

## Author summary

*Klebsiella pneumoniae* is a major cause of health care–associated infections worldwide, and infections with hv*Kp* is often associated with cytokine storms and lymphopenia, resulting in higher mortality. However, the underlying mechanisms of lymphopenia during hv*Kp* infection is not fully understood. In this study, we demonstrate a mechanism of immunosuppression of T cells in hv*Kp* infection mouse model by combination of single-cell RNA sequencing, flow cytometry and untargeted metabolomic analysis. We confirm MDSCs can suppress T cell proliferation and induce T cell apoptosis by activating tryptophan metabolism during hv*Kp* infection. Specifically, the downstream metabolite kynurenine, which catalyzes by IDO1 in tryptophan metabolism pathway, serves as the signaling molecule in the interactions between MDSCs and T cells. Our findings provide a new insight in understanding the mechanism of how hv*Kp* manipulate the host immune systems and suggest that pharmacological inhibition of IDO1 could be an effective therapeutic strategy for combating hv*Kp* infection.

## Introduction

*Klebsiella pneumoniae* (*Kp*) is a Gram-negative, non-motile, and usually encapsulated bacillus belonging to the *Enterobacteriaceae* family. It is an opportunistic pathogen that can cause both hospital-acquired and community-acquired infections [1]. In hospitals, *Kp* is responsible for both endemic infections and outbreaks of epidemic strains, and it is the main cause of neonatal sepsis [2,3]. While it often starts as a localized infection or colonization in the urinary tract, GI tract, or respiratory tract, it can spread to the bloodstream and result in sepsis [4]. Although there have been significant advancements in clinical management, recent reports suggest that outcomes have only a slightly improvement. The initial stage of septic shock is marked by a systemic hyperinflammatory immune response [5], which can lead to organ failure and early mortality. However, if patients survive this initial assault, they enter a state of immune deactivation state known as compensatory anti-inflammatory response syndrome. Our previous study revealed that hypervirulent *Kp* (hv*Kp*) induced mortality was associated with a cytokine storm and lymphopenia^6^. Previous studies has linked T cell dysfunction to sepsis mortality [6,7] and increased susceptibility to secondary infections [8]. However, the mechanisms underlying hv*Kp* infection induced lymphopenia are still not fully understood.

Several mechanisms, such as apoptotic depletion of immune cells, increased expression of negative costimulatory molecules, increased regulatory T cells (Tregs), expression of programmed cell death (PD)-1 on CD4 T cells [9], and the emergence of myeloid-derived suppressor cells (MDSC) [10], contributed to bacteremia-induced immunosuppression. Interestingly, mechanisms involved in MDSC-mediated T cell dysfunction shared some features with those described in malignancies, including arginase-induced arginine depletion and its impact on T cell mitochondrial dysfunction and enhanced apoptosis [11,12]. Additionally, MDSC activation also leads to a high expression of amino acid metabolization enzymes, contributing to immunosuppressive properties of MDSCs [11]. Access to amino acids is crucial for naïve T cells to proliferate and differentiate into effector cells. However, T cells have differential requirements for specific amino acids.

Depletion of tryptophan (Trp) or arginine (Arg) activates general control non-derepressible 2 (GCN2) and blocks entry into S phase following T cell stimulation, while depletion of isoleucine and leucine leads to cell cycle entry, and subsequent cell death [13]. It also has been found that idoelamine 2,3-dioxygenase (IDO) is highly expressed in monocytic-MDSC (M-MDSC) in chronic lymphocytic leukemia, which catabolizes the rate-limiting step of the kynurenine (Kyn) pathway, resulting in lower Trp levels and the accumulation of Kyn within the tumor microenvironment (TME) [14]. This depletion of Trp and accumulation of Kyn suppress T cell activation and induce Treg *in vitro* [15]. Furthermore, IDO inhibition has been shown to reduce the tumor infiltration of MDSCs and inhibit their suppressive character [16], indicating the crucial role of IDO1-mediated tryptophan metabolite in the suppression of T cell-mediated immune response. Using a mouse bacteremia model with hv*Kp* infection, we conducted a comprehensive analysis of MDSC metabolism and its impact on T cell function in hv*Kp*-infected lungs. We found that reducing the frequency of MDSCs through *Ido1* deficiency was effective against hv*Kp* infection, suggesting that inhibiting IDO1 could be a potential therapeutic approach to mitigate *Kp*-induced mortality.

## Result

### scRNA-seq reveals gene expression signature of CD4 T cells in 17ZR101-infected mice

**Fig 1:**
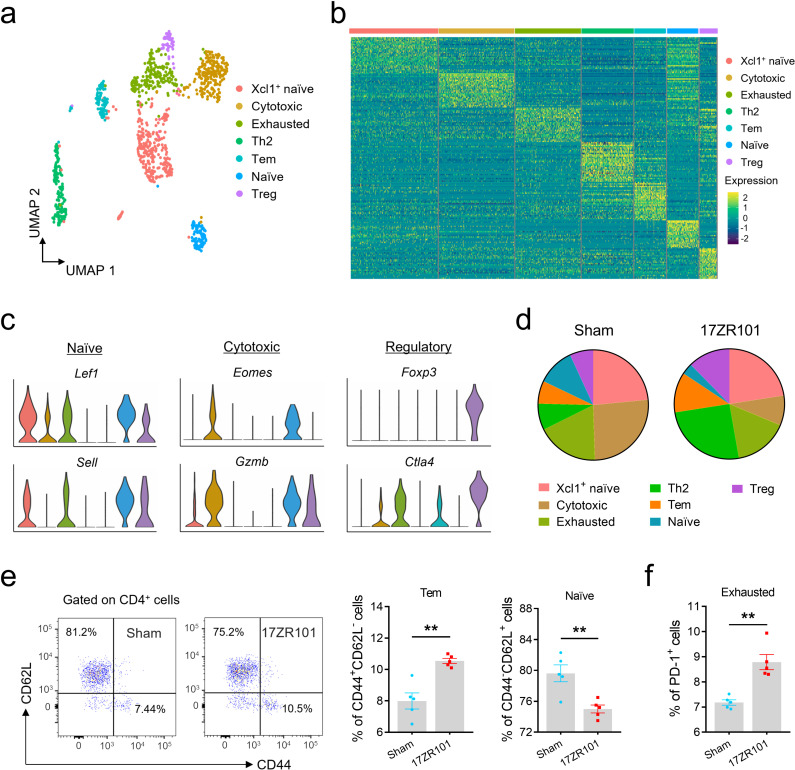
CD4 T cell subset composition in 17ZR101-infection. (a) UMAP projection of all 1,063 CD4 T cells presenting seven different subsets, identified via shared nearest neighbor modularity optimization-based clustering algorithm, followed by merging of similar clusters. (b) Heatmap of gene expression z scores across cells. All CD4 T cells were grouped by subset (horizontal bars). Genes shown were up-regulated significantly in at least one subset compared to all other cells. Genes were ordered by significance and associated with the subset with higher detection rates. (c) Violin plots showing the expression (z score) of selected canonical marker genes across all seven subsets. (d) Representative pie charts showing the percentage of cells belonging to each of the seven subsets in a sham-infected mouse and a 17ZR101-infected mouse. (e) Representative dot plots and quantification of Naive and Tem CD4 T cells in Sham- and 17ZR101-infected lungs at 12 hpi. n=5/group. (f) Exhausted CD4 T cells recovered from *Kp*-infected lungs were analyzed by flow cytometry. n=5/group. Data was represented as mean ± SEM. **p < 0.01. *P* values were derived from the unpaired two-tailed Student’s t-*t*ests (e and f).

In our previous study in which we were trying to investigate the underlying mechanisms of hv*Kp* pathogenesis, we employed a hv*Kp* strain 17ZR101 in the study. This strain was recovered from a 43-year-old female patient in the ICU and was tested hypermucoviscous phenotypically. We found that mice infected by 17ZR101 died within 24 hours post-infection (hpi) with a series of tissue damages in various organs. In addition, 17ZR101-infection resulted in significantly higher bacterial burdens in various organs at 12 hpi compared with classical *Kp*-infected mice [17]. Single-cell RNA Sequencing (scRNA-Seq) analysis revealed that mice infected with the hv*Kp* strain 17ZR101 died from cytokine storm and also experienced lymphopenia [17]. One of the mechanisms responsible for immunosuppression is the increased expression of negative costimulatory molecules on CD4 T cells [9]. To identify the underlying mechanism of lymphopenia and classify CD4 T cell subsets in an unbiased manner, we clustered cells based on their transcriptomic profiles and assessed the robustness of the clusters’ identity. By combining all cells from Sham- and 17ZR101-infected mice, we identified 7 distinct clusters of CD4 T cells (Fig 1a). To associate each cluster with a known CD4 T cell subset, we screened for the most significantly up-regulated genes (combined *P* < 10^−3^) in each cluster and compared them to previously reported T cell subsets and canonical markers (Fig 1b and 1c). Out of the seven distinct clusters, three matched established subsets: a population of naïve T cells overexpressing *Lef1*, *Sell*, and *Igfbp4* genes (referred to as ‘Naïve’); a population of Tregs, identified by their classical expression of the *Foxp3* gene, along with the expression of naïve-associated genes *Lef1* and *Sell* [18]; and cells overexpressing genes such as *Eomes* and *Gzmb*, which are commonly associated with CD8 T cells (denoted ‘Cytotoxic’) and have been previously described as CD4 cytotoxic T cells in the context of viral infection and cancer [19]. We also observed a population of naïve CD4 T cells (denoted ‘Xcl1^+^ naïve’) expressing *Lef1* and *Sell* genes along with *Xcl1*, which is a ligand of XCR1 (or G protein-coupled receptor 5, GPR5), with increased expression on NK and CD8^+^ T cells during virus infection. Additionally, we found cells with an exhaustion signature (denoted ‘Exhausted’) that overexpressed the *Tnfsf4* and *Tnfsf8* genes [20,21]. These cells were highly enriched for *Ctla2a*, *Il5*, and *Il1rl1*, indicating of a Th2 cell phenotype [22]. It has been reported that the shift from a Th1 to a Th2 cell profile contributes to sepsis-associated immune dysfunction [23], and effector-memory T cells (Tem) expressing the *Igals3* and *S100a4* genes [24].

Next, we compared the proportion of different subsets in mice infected with 17ZR101- versus Sham. We found that the Xcl1^+^ naïve subset and Exhausted T cell subset had similar abundances in both groups; whereas the naïve and cytotoxicity subset were predominant in the Sham-infected group, while the Th2, Tem, and Treg subsets were dominant in 17ZR101-infected mice (Fig 1d). We then performed flow cytometry to analysis the naïve (CD44^-^CD62L^+^), Tem (CD44^+^CD62L^-^) and exhausted (PD-1^+^) CD4 T cells from infected lungs. Compared with Sham-group, 17ZR101-infection resulted in an increased frequency in Tem and exhausted CD4 T cells, but with a decreased naïve population (Fig 1e and 1f). Overall, these findings indicate that 17ZR101-infection leads to a complex landscape of CD4 T cells. Importantly, the frequency of Tem was associated with elevated levels of inflammatory cytokines in the sera (primarily interleukin (IL)-1β and IL-6) [17], suggesting a potential link between the process of inflammation [25] and the fate of CD4 T cells in 17ZR101 infection.

### T cell apoptosis and T-cell proliferation inhibition result in lymphopenia

**Fig 2:**
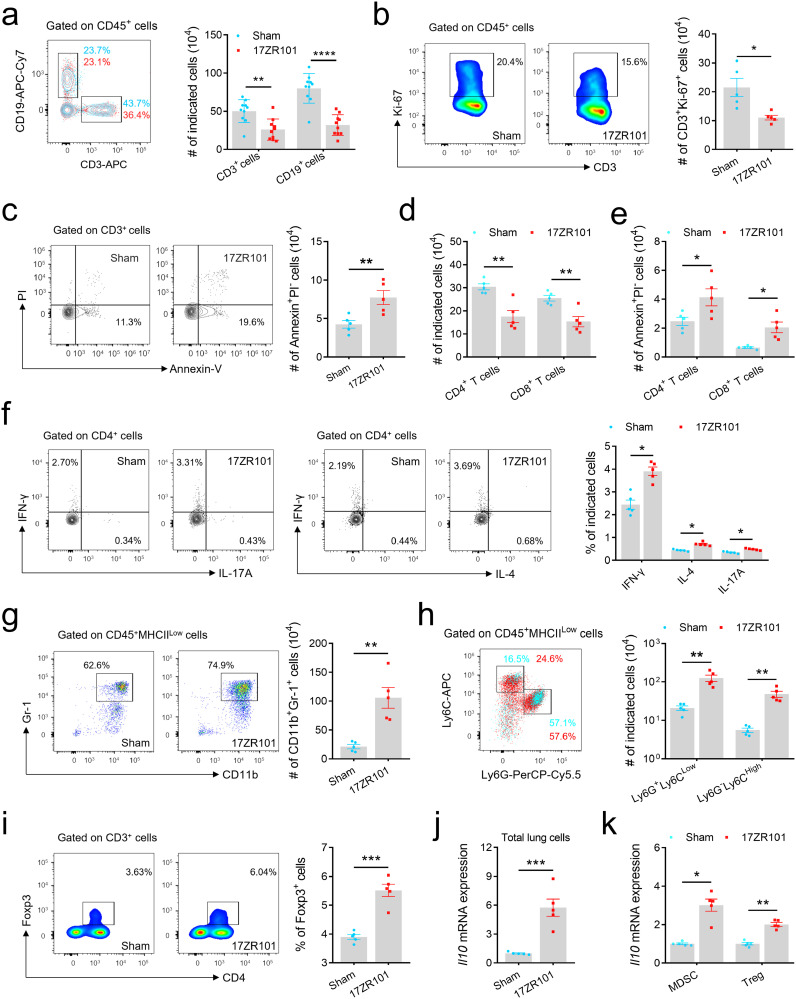
Lymphopenia was associated with impeded proliferation and enhanced apoptosis in 17ZR101-infected lungs. C57BL/6 mice were inoculated with 10^4^ CFU of hv*Kp* strain 17ZR101 intravenously and infected-lung samples were collected for analysis. (a) Lymphocytes recovered from *Kp*-infected lungs were analyzed by flow cytometry. n=5/group. (b) Flow cytometry plots of Ki-67 expression following 17ZR101 infection in CD3^+^ cells. (c) Flow cytometry detection of T cell apoptosis with Annexin V/PI test. A Representative of flow cytometry with annexin-V/PI plots in Sham- and 17ZR101-infected lungs at 12 hpi. (d) Quantification of CD4 T cells and CD8 T cells in Sham- and 17ZR101-infected lungs at 12 hpi. (e) Flow cytometry detection of CD4 and CD8 T cell apoptosis with Annexin V/PI test. (f) Total lung cells from sham- and 17ZR101-infected mice were cultured with activation cocktail. After 4 h of culture, cells were harvested and intracellular staining was performed (IFN-γ, IL-4 and IL-17A), and cytokine-producing CD4 T cells were analyzed by flow cytometry. (g) Flow cytometry analysis showing percentages of CD11b^+^Gr-1^+^ cells among total lung cells and representative bar diagrams showing numbers of CD11b^+^Gr-1^+^ cells in the lung at 12 hpi. (h) Representative dot plots showing percentages of M-MDSC and PMN-MDSC within CD11b^+^ cells in Sham- and 17ZR101-infected lungs at 12 hpi. (i) Representative bivariate flow cytometry plots showing Foxp3 expression on live CD4 T cells obtained from matched lung samples. (j) Fold-change in expression level of *Il10* in lungs of 17ZR101-treated mice compared with lung cells of Sham-infected mice. n=5. (k) Fold-change of *Il10* expression level in MDSC and Tregs from lungs of 17ZR101-treated mice compared with lung cells of Sham-infected mice. n=5. Data was represented as mean ± SEM. NS, not significant; *p < 0.05, **p < 0.01, ***p < 0.001, ****p < 0.0001. *P* values were derived from unpaired two-tailed Student’s *t*-tests (b, c, g, I and j) and two-way ANOVA wi*t*h Tukey’s multiple-comparison test (a, d, e, f, h and k).

The flow-cytometry analysis was performed to confirm the lymphocyte compartment revealed by scRNA-Seq. We observed a significant decrease in the total cell number of T cells (CD3^+^), and B cells (CD19^+^) in 17ZR101-infected lungs, confirming the presence of lymphopenia after the infection (Fig 2a), which was confirmed in a murine pneumoniae model (S1 Fig). Previous studies have shown that many patients who died of sepsis had unresolved opportunistic infections [26] and immunosuppressive features [9]. First, we investigated whether this lymphopenia was caused by impaired T cell proliferation or lymphocyte apoptosis. Our results demonstrated an immunosuppressive feature with reduced proliferation (Fig 2b) and increased apoptosis (Fig 2c), both contributing to the reduction of T cell. Specifically, we observed both CD4 and CD8 T cells were undergoing the higher level of apoptosis (Fig 2d), resulting the decreased number in CD4 and CD8 T cells (Fig 2e). Another characteristic of lymphopenia during chronic viral infections is the failure to produce effector cytokines [27]. Therefore, we examined whether the lymphopenia in the 17ZR101-infection led to impaired induction of peripheral antigen-specific CD4 effector T cell responses. To test this, we isolated total lung cells from Sham- and 17ZR101-infected mice after 12 hours of injection and incubated them with an activation cocktail to measure antigen-specific cytokine production. We observed significant increases in the frequency of CD4^+^IL-17^+^ and CD4^+^IFN-γ^+^ T cells, but no differences in CD4^+^IL-4^+^ T cells between these two groups, without any deficiency in CD4 cytokine production (Fig 2f).

Because Tregs and MDSCs are major components of the immune suppressive microenvironment. MDSC-mediated suppression of host immunity is crucial for immune regulation and tolerance during chronic inflammation, but it can be detrimental in other pathologic disorders and infections. In order to determine the presence of MDSCs in 17ZR101 infection, we compared the frequencies of CD11b^+^Gr-1^+^ MDSCs in the lungs of healthy control subjects and subjects exposed to hv*Kp*. We observed a large population of MDSC expansion (Fig 2g), with an enlarged population of both M-MDSC and G-MDSC in infected lungs (Fig 2h). A previous study identified that monocytic MDSCs (CD11b^+^Gr-1^+^CD115^+^) induced the activation of Foxp3^+^ Tregs [28]. Consistently, CD4^+^Foxp3^+^ Tregs accounted for approximately 6% of total CD4 cells in 17ZR101-infected lungs compared to 3.6% in sham-infected lungs (Fig 2i), suggesting a link between MDSCs and Tregs in T cells suppression.

As immature myeloid cells obtained from tumor-bearing hosts are known to be immunomodulatory^29^ [29], we also examined whether cells obtained from bacteremic mice could produce inflammatory mediators, including IL-10, a cytokine generally regarded as necessary for T cell suppression. We found that 17ZR101-infection was associated with higher *Il10* mRNA expression in infected lungs (Fig 2j), especially in infected Tregs and MDSCs (Fig 2k). Taken together, these results suggest that 17ZR101 infection causes T cell immunosuppression in mice.

### The inhibition property of MDSC is associated with Trp metabolism in 17ZR101-infected lungs

**Fig 3:**
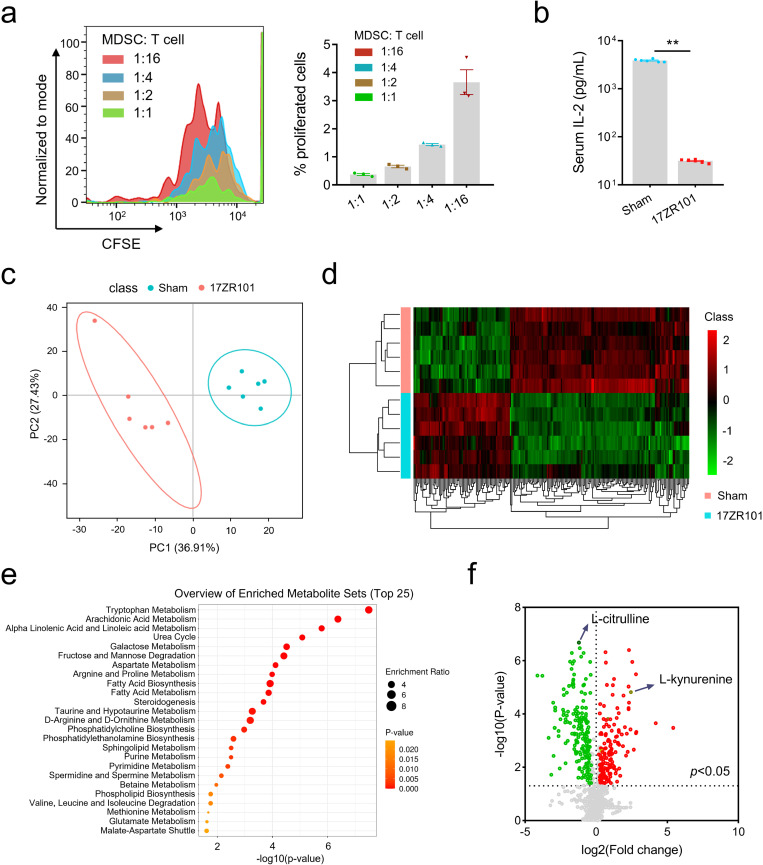
MDSC inhibition was correlated with Trp metabolism in 17ZR101-infected lungs. C57BL/6 mice were inoculated with 10^4^ CFU of hv*Kp* strain 17ZR101 intravenously and the Gr-1^+^ cells were isolated at 12 hpi for T cell inhibition analysis. (a) CFSE-labeled normal spleen T cells were stimulated with immobilized anti-CD3e and anti-CD28 mAb for 3 days in the presence of Gr-1^+^ cells (ratio: 1:1, 1:2, 1:4 and 1:16). Proliferation of T cells was evaluated as CFSE dilution by flow cytometry. (b) Quantification of serum IL-2 level from Sham- and 17ZR101-infected mice measured by ELISA kit. (c) Partial least square discriminant analysis (PLS-DA) scores plot representing lung metabolites altered between the 17ZR101-infected (red) and Sham-infected group (green). (d) Heatmap of metabolic concentrations in lungs obtained from uninfected C57BL/6 mice or 17ZR101-infected mice subjected to metabolomic analysis (n=6 mice/group). (e) KEGG pathway-based quantitative enrichment analysis indicating the most affected metabolic pathways in hv*Kp* infection. (f) Volcano plot analysis of the significantly increased or decreased metabolites. n=6/group. Fold change > 1.2 and FDR-adjusted P-value < 0.05 considered significant. Mean ± SEM. is represented in the data. **p < 0.01. *P* values were derived from unpaired two-tailed Student’s *t*-tests (b).

In order to assess the suppressive function of MDSCs, we first examined their ability to inhibit the proliferation of CD3^+^ T cells *ex vivo*. MDSCs from infected mice were co-cultured with CD3^+^ T cells, and the proliferation of T cells was assessed after three days of culture. Our findings indicate that MDSCs significantly impede the proliferation rate of CD4 cells (Fig 3a). IL-2 is a central cytokine released by T cells that promotes both T cell proliferation and differentiation. Analysis of IL-2 levels in co-cultured medium revealed a correlation between the concentration of serum IL-2 and the dose-dependent inhibition of T cell proliferation by MDSCs (Fig 3b). Multiple inhibitory mechanisms, including IL-10, transforming growth factor-β (TGF-β), and arginase-1, have been associated with MDSC-mediated suppression of T cell effector function by depleting extracellular L-Arg, Trp and NO.

To investigate the potential contribution of metabolic changes to lung pathology and uncover the underlying MDSC metabolism within the bacteremia microenvironment, we conducted an untargeted metabolomics analysis of lung tissues from Sham- and 17ZR101-infected mice. The results of the PLS-DA analysis revealed a clear distinction between the two groups, indicating significant metabolic differences (Fig 3c). To validate the predictive ability of the PLS-DA model, we performed leave-one-out cross-validation (LOOCV) and obtained a Q2 score of 0.67, indicating a high-quality of the PLS-DA model [30]. Our model demonstrated good fit and a predictive ability. The overall metabolome associated with 17ZR101 infection differed significantly from that of the Sham-infection group (Fig 3d). Out of the 1,260 evaluated metabolites, 341 showed significant differences with a fold change greater than 20% between the two groups. Among the top 25 pathways, tryptophan metabolism exhibited the most pronounced alterations (Fig 3e). Further analysis using a volcano plot revealed a significant decrease of L-Kyn from the Trp metabolism pathway in the 17ZR101-infected group compared to the Sham group (Fig 3f). Additionally, L-citrulline from the Arg metabolism pathway was found to significantly increase with 17ZR101-infection (Fig 3f). Taken together, these findings suggest a correlation between MDSC and Trp metabolism in 17ZR101-infected lungs.

#### L-Kyn inhibits T cell proliferation and induces T cell apoptosis.

Since the discovery the immunosuppressive effects of MDSCs, there are increasing evidences supporting the crucial role of IDO1 in immune regulation [31]. The levels of free Trp in the body are influenced by food intake and various Trp metabolizing pathways. While only a small portion of free Trp is used for protein synthesis and the production of neurotransmitters like serotonin and neuromodulators such as tryptamine [32], more than 95% of free Trp serves as a substrate for the Kyn pathway of Trp degradation. This pathway produces several metabolites with distinct biological activities in the immune response and neurotransmission [33]. Additionally, the increased expression of IDO1 during Trp metabolism suggests IDO1 may have a previously unrecognized role in 17ZR101-infection (S2 Fig).

**Fig 4:**
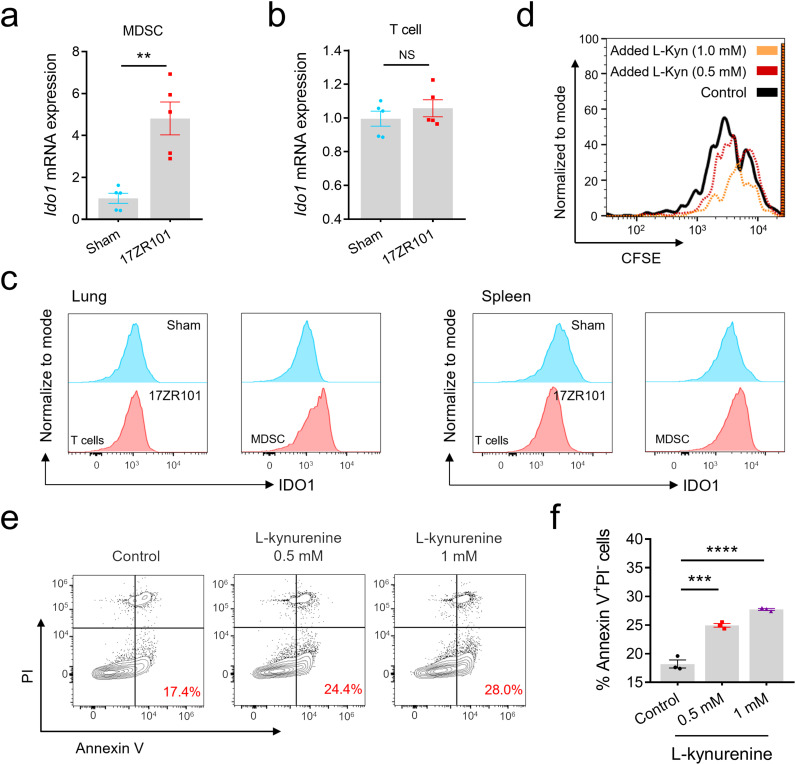
PMN-MDSCs suppressed T cell proliferation through L-kyn. C57BL/6 mice were inoculated with 10^4^ CFU of hv*Kp* strain 17ZR101 intravenously and the Gr-1^+^ cells and T cells were isolated at 12 hpi for RNA isolation and RT-qPCR analysis. Fold-change in expression level of *Ido1* in MDSCs (a) and T cells (b) of 17ZR101-infected mice, with lung cells of Sham-infected mice as control. n=5. (c) The flow cytometry analysis of IDO1 expression in T cells and MDSCs in Sham- and 17ZR101-infected lungs and spleens. (d) Splenic CD3 T cells were CFSE-labelled and stimulated with anti-CD3e and anti-CD28, with added indicated concentration of kynurenine. After three days, proliferation was assessed by flow cytometry. Experiment representative of six independent replicates. (e, f) Splenic T cells were co-stimulated using CD3/CD28 mAb coated antibodies for three days, with added indicated concentration of kynurenine. After three days, apoptosis was assessed by flow cytometry. Data was represented as mean ± SEM. NS, not significant; **p < 0.01, ***p < 0.001, ****p < 0.0001. *P* values were derived from unpaired two-tailed Student’s *t*-tests (a and b) and one-way ANOVA followed by Tukey’s correc*t*ion for multiple comparisons (f).

To investigate the role of IDO1 in suppressive ability of MDSC, we evaluated the expression of *Ido1* mRNAs in purified PMN-MDSC from both Sham- and 17ZR101-infected animals. Our results showed that PMN-MDSC but not T cells from 17ZR101-infected animals expressed a higher level of *Ido1* mRNA compared to Sham-infected animals (Fig 4a and 4b). This suggests that the activation of Trp metabolism occurs in MDSC. Trp degradation is known to suppress immune cells through the formation of immunosuppressive Trp catabolites and depleting Trp itself [33]. A severe shortage of Trp can lead to dysfunction of T cells and antigen-presenting cells by inducing the GCN2 kinase pathway [13]. However, even when dioxygenase expression is forced, Trp levels in the local microenvironment do not fall to a level sufficient to activate GCN2. Additionally, previous studies have shown that Kyn/Trp ratios can serve as early indicators of the development of sepsis [34,35]. Therefore, we hypothesized that the immunoregulatory properties of Trp metabolism are mainly a consequence of its metabolites rather than the depletion of Trp in 17ZR101-infected lungs. This led us to question the interaction between Trp metabolism in MDSC and T cells. We observed L-Kyn strongly suppressed T cell proliferation in a dose-dependent manner (Fig 4c), also with significant increased cell apoptosis (Fig 4d and 4e). Our observations confirmed that MDSC-mediated Trp metabolism impairs T cell proliferation and induces T cell apoptosis through the increased level of L-Kyn. Overall, these results demonstrate that the increased population of MDSC in 17ZR101-infected lungs suppresses T cell proliferation, which is a result of L-Kyn generated by Trp metabolism.

### IDO1 inhibition combates 17ZR101-infection by reversing lymphopenia

**Fig 5:**
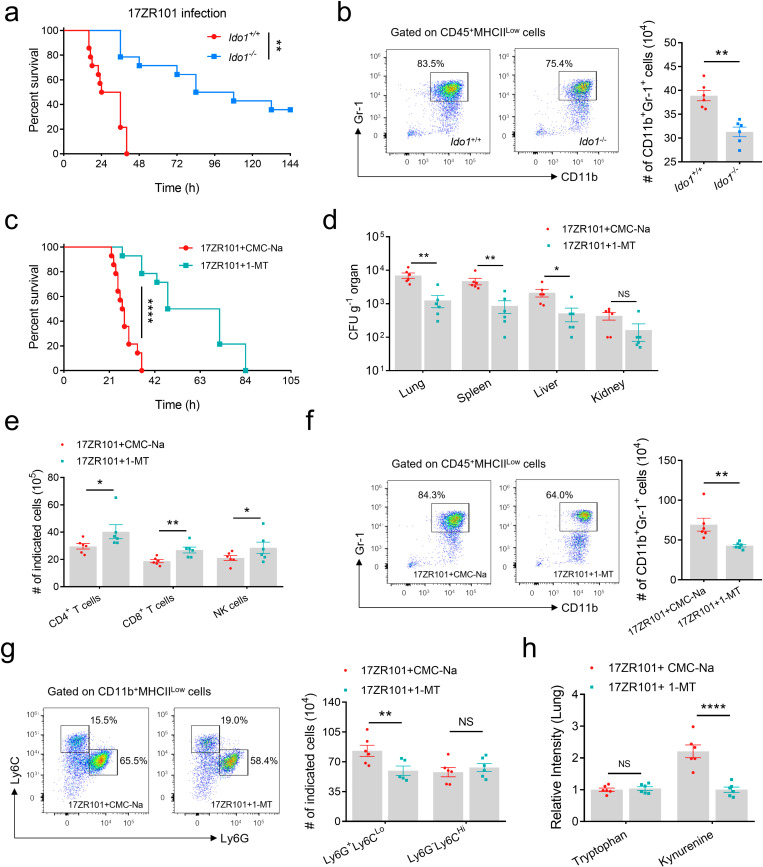
*Ido1* knockout or inhibition improves survival in 17ZR101 infection. (a) Kaplan-Meier survival curves of *Ido1*^-/-^ and *Ido1*^+/+^ mice were inoculated with 10^4^ CFU of indicated K2-hv*Kp* (17ZR101) strain intravenously. n = 14/group. (b) Representative dot plots showing percentages of MDSC within CD45^+^ cells and representative bar diagrams showing numbers of MDSCs in the *Ido1*^+/+^ and *Ido1*^-/-^ lungs. n = 6/group. (c) Kaplan-Meier survival curves of mice treated with IDO1 inhibitor 1-MT and mice were inoculated with 10^4^ CFU of hv*Kp* strains. n = 14/group. (d) C57BL/6 mice was treated with 1-MT and then challenged with 17ZR101 intravenously. Bacterial load in various organs of mice was measured at 12 hpi. n = 6/group. (e) Lymphocytes recovered from *Kp*-infected lungs were analyzed by flow cytometry and quantification of CD4, CD8 T cells and NK cells. n = 6/group. (f) Representative dot plots showing percentages of CD11b^+^Gr-1^+^ cells among total lung cells and representative bar diagrams showing numbers of MDSCs in the lung. n = 6/group. (g) Representative dot plots showing percentages of M-MDSC and PMN-MDSC within CD11b^+^ lung cells with or without 1-MT treatment. (h) The relative intensity of Trp and Kyn in lungs of Sham- and 17ZR101-infected mice with or without 1-MT treatment. n=6. Data was represented as mean ± SEM. NS, not significant; *p < 0.05, **p < 0.01, ****p < 0.0001. *P* values were derived from the log-rank test (a and c), unpaired two-tailed Student’s *t*-tests (b and f) and *t*wo-way ANOVA with Tukey’s multiple-comparison test (d, e, g and h).

The rate-limiting step in the Kyn pathway is the enzymatic conversion of Trp to N-formyl kynurenine by IDO1, IDO2 and tryptophan-2,3-dioxygenase (TDO), and depletion of Trp by these enzymes can have fundamental consequences on cellular function and survival [33]. The expression of IDO in healthy tissues is generally quite low but is markedly upregulated in response to infection and inflammation. Recent studies have established an entirely novel important biological function for IDO. These studies indicate that IDO-mediated tryptophan depletion *in vitro* results in inhibition of matrix-degrading metalloproteinase enzyme production, suppression of inflammatory responses, and promotion of immune tolerance [36]. Then we generated *Ido1* knockout mice to test whether *Ido1* could be a target in *Kp* infection. We observed that IDO1 knockout strongly reversed the severe mortality caused by 17ZR101 infection (Fig 5a). Convincingly, T cell suppression was attenuated with less infiltrated MDSC in infected *Ido1*^-/-^ mice (Fig 5b).

To further confirm the role of IDO1 in the pathogenesis of hv*Kp* infection, mice were orally given 1-Methyl-D-tryptophan (synonyms: indoximod; 1-D-MT/1-MT, an effective inhibitor of IDO1) at 36 h and 12h before infected with hv*Kp* strain 17ZR101. Our results showed that 1-MT treatment could protect 50% of mice from death due to 17ZR101 infection at 60 h post infection (Fig 5c). Consistent with this finding, we observed that IDO1 inhibition showed a reduction in various organ CFU after challenged with 17ZR101 (Fig 5d). Accordingly, 1-MT treatment reversed the immune cell composition induced by 17ZR101-infection, with recovered T lymphocyte and decreased MDSC, particular PMN-MDSC, in infected lungs and spleens (Figs 5e-g and S3). To make it more convincing, we tested the trp and Kyn level in 1-MT-treated mice. We observed that the lower level of kyn in 1-MT inhibited serum, but not in CMC-Na-treated group (Fig 5h). Together, these results showed that IDO1 targeting could be a therapeutic strategy in hv*Kp*-induced murine bacteremia.

## Discussion

The hv*Kp* strain, encapsulated with CPS, has been well-documented to evade neutrophil-mediated killing by avoiding phagocytosis *in vitro* [37,38]. This ability allows *Kp* to resist destruction and trigger a cytokine storm, potentially leading to host mortality [17]. In our study, we demonstrated that 17ZR101-infection induced the accumulation and activation of MDSCs, particularly PMN-MDSCs, known for suppressing T-cell responses [11]. Previous research has shown similar MDSC accumulation and immune-suppressive activity following infections with *M. tuberculosis* [39], hepatitis C virus [40] and parasites [41]. However, the role of MDSCs in *Kp* infected lungs remains controversial. One study showed a decrease in M-MDSCs after KPPR1 infection [42], while another reported that early recruitment of M-MDSCs to the lungs during CR-*Kp* (ST258, KP35) infection promoted bacterial clearance, protected lung tissue, and improved host survival through IL-10 production [43]. Additionally, MDSC-like cells recruited during KPPR1 infection appeared to aid in clearing apoptotic neutrophils via efferocytosis, producing IL-10 to enhance lung recovery [44]. Some studies suggest that immunosuppressive M-MDSCs contribute to the emergence of the ST258 strain KP35 as a pulmonary pathogen by reducing phagocytic efficiency without aiding bacterial clearance [45]. Wong *et al*. reported that ST258 strain MKP103 infection promotes tolerance to pulmonary infection through G-MDSC recruitment [46]. Comparing these studies, we speculate that IL-10 production by early recruited M-MDSCs is crucial for directing the immune response and ensuring host survival. Rapid IL-10 production prevents uncontrolled cytokine release, reduces lung damage, and improves bacterial clearance, albeit with delayed airway clearance. In our study, hv*Kp* strain 17ZR101 infection induced the tolerogenic activity of MDSCs (Fig 2g), exacerbating the infection. However, the expansion of M-MDSC and G-MDSC was inversely correlated with T-cell counts, resulting in better bacterial survival. This immunosuppression and imbalance between innate and adaptive immune responses delay pathogen clearance and promote lung injury. The kinetics of IL-10 production and its cellular source were not detected, warranting further investigation in future studies.

MDSCs play a crucial role in regulating immune responses in various conditions, including anti-tumor and anti-sepsis responses. They achieve this through both antigen-specific and antigen-non-specific mechanisms, employing multiple strategies to regulate immune responses and inhibit Teff [47]. In peripheral lymphoid organs, MDSCs suppress CTLs by mediating antigen presentation and directly interacting with T cells [48]. However, they can also suppress nearby T cells in an antigen-independent manner at tumor sites and in the periphery. MDSCs confer immunosuppression by depleting nutrients, such as consuming L-arginine through Arg1-dependent mechanisms or depleting L-cysteine by sequestering it [49], leading to the arrest of antigen-activated T cell proliferation [50]. Additionally, MDSC-derived IDO consumes L-Trp, resulting in the accumulation of Kyns, which further inhibit T cell activation [33]. Notably, increased IDO activity has been observed in COVID-19 patients at admission and is associated with disease severity [51]. In our study, we found that Trp metabolism is a prominent feature of the lung microenvironment during 17ZR101 infection. Specifically, there was no difference in Trp concentration between Sham- and 17ZR101-infected lungs and serum. As Trp concentrations are also influenced by dietary intake. One possible mechanism for the unchanged tryptophan concentration is that a shortage of amino acids inactivates mTOR, inducing autophagy and decreasing protein synthesis. This recycling of amino acids helps maintain their systemic concentration [52]. However, we observed a significant increase in Kyn concentration during *Kp* infection, with higher *Ido1* expression in 17ZR101-infected MDSCs (Fig 4a). IDO1 was induced during systemic inflammation, as confirmed by studies on patients receiving a known amount of tryptophan through TPN [53]. Furthermore, studies have indicated that significantly increased Kyn-Trp ratios can predict subsequent sepsis, multiple organ failure, and death [53]. Both increased Kyn values and Kyn-Trp ratios could potentially serve as predictors for the posttraumatic development of sepsis and organ failure.

In a recent study, exploring Kyn as a potential immunosuppressive metabolite for supplemental therapy, these authors find that L-kyn demonstrated immunosuppressive effects *in vivo*, however, the concentration of Kyn in human tumors was only in the low micromolar range, which is far below the concentration required to induce T cell apoptosis *in vitro* [54]. They speculated that the inefficacy of IDO inhibitors in human cancer clinical trials was due to tissue levels of kynurenine and its derivatives that were too low to act as a major metabolic barrier to anti-tumor immunity. In this study, we used the IDO1 inhibitor 1-MT to reduce the concentration of L-kyn in the microenvironment as a potential treatment for hv*Kp* infection (Fig 5c), which was confirmed in *Ido1*^-/-^ mice (Fig 5a). To eliminate strain-specific bias, we infected *Ido1*^-/-^ mice with different serotypes of hv*Kp* strains using an intraperitoneal infection model. The results indicated that IDO1 serves as a general therapeutic target for hv*Kp* infection (S4 Fig). This finding suggests the potential clinical significance of targeting IDO1 for the treatment of hv*Kp*-induced bacteremia.

In this study, multiple infection models were employed to investigate the effects of hv*Kp* infection. The intranasal route was utilized to ensure that the infection remained primarily localized to the lungs, the target organ in pneumonia. In contrast, the intravenous infection model resulted in an almost immediate systemic infection, as the bacteria were rapidly disseminated throughout the body via the circulatory system. This widespread distribution can complicate the interpretation of results and hinder the study of lung-specific responses. By combining intranasal (S1 Fig) and intravenous infection models, we confirmed that hv*Kp* infection led to lymphopenia in a murine model. Additionally, while intraperitoneal infection can also induce systemic infections if the bacteria spread from the peritoneal cavity, recent research has demonstrated that intravenous infection with *Nocardia farcinica*, compared to intranasal and intraperitoneal routes, induces significant clinical symptoms, triggers an inflammatory response, damages multiple organs, and leads to systemic infections [55]. Subsequently, we infected *Ido1*^-/-^ mice with hv*Kp* strains intravenously (Fig 5a) and intraperitoneally (S4 Fig) and found that IDO1 knockout conferred protection to mice in against hv*Kp*-induced systemic infection.

IDO1 is the initial rate-limiting enzyme in the Trp metabolic pathway. Dysregulation of Kyn and its downstream metabolites, which leads to an imbalance in Kyn metabolite levels in the brain, has been associated with neurodegenerative conditions and psychological disorders, including schizophrenia and depression [56]. Studies have shown that wild-type mice under inflammatory conditions exhibit increased anxiety-like behavior in open field and light/dark transition tests, heightened depressive-like behavior in forced swim and tail suspension tests, and reduced novel object recognition. In contrast, *Ido1* knockout mice do not display these behaviors [57,58]. However, in this study, *Ido1* knockout mice demonstrated greater resistance to *Kp* infection (Fig 5a), a finding that did not account for the effects between these metabolites on brain function and anxiety-like behaviors and *Kp* infection. The relationship between these metabolites in the Trp metabolic pathway, their role in brain development and function, and their impact on *Kp* infection warrant further investigation.

**Fig 6:**
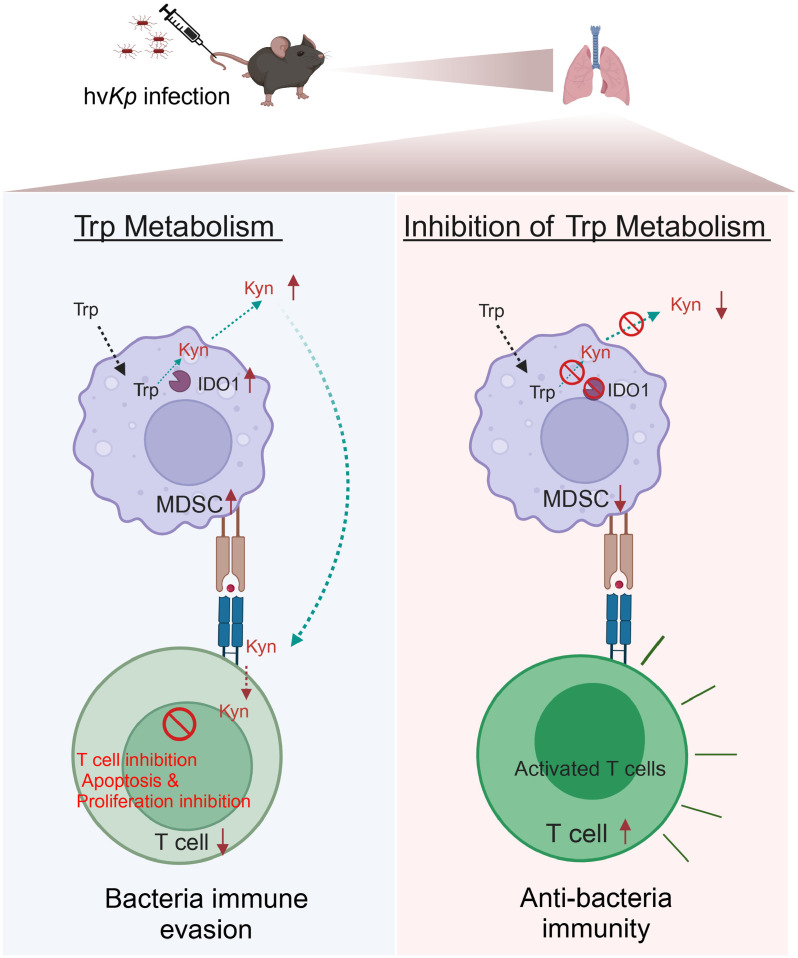
Mechanism of MDSC suppresses T cell-based immunity against hv*Kp* infection. In hv*Kp* -infected lungs, MDSCs inhibit T-cell proliferation, resulting in lymphopenia, which further facilitates bacterial immune escape. L-Kyn in MDSCs, a product of tryptophan metabolism catalyzed by IDO1, inhibits T-cell proliferation and induces T-cell apoptosis. This suppression hampers T-cell-mediated responses against hv*Kp*. Inhibition or knockout of IDO1 could reverse lymphopenia and enhance the T-cell-mediated responses against hv*Kp* (Created in BioRender. Xu, Q. (2025) https://BioRender.com/t97j916”.).

To conclude, MDSCs inhibit T-cell proliferation, resulting in lymphopenia, which further facilitates bacterial immune escape in hv*Kp*-infected lungs. L-Kyn in MDSCs, a product of tryptophan metabolism catalyzed by IDO1, inhibits T-cell proliferation and induces T-cell apoptosis. This suppression hampers T-cell-mediated responses against hv*Kp* (Fig 6). Our results revealed a previously unrecognized pathway involved in hv*Kp* induced septic shock, the modulation of which may present novel therapeutic strategies and targets to treat hv*Kp* infection.

## Method

### Ethics statement

The use of mice was approved by the Animal Ethics Committee of the City University of Hong Kong (#A0421) and The Hong Kong Polytechnic University Shenzhen Research Institute and followed the guidelines of the Institutional Laboratory Animal Research Unit (#23-24/843-FSN-R-NSFC).

### Mice

Six- to eight-week-old animals of both sexes were used in this study. WT C57BL/6 and *Ido1*^-/-^ [C57BL/6NCya-*Ido1*^*em1*^/Cya, S-KO-02565] mice were bred in-house or purchased from Cyagen. All mice were housed and bred under specific pathogen-free conditions. All experiments were performed using sex- and age-matched controls.

### Bacteria strain

*Kp* strain 17ZR101 (ST86, K2) was recovered from a 43-year-old female patient in the ICU of The Second Affiliated Hospital of Zhejiang University in 2017. The patient had undergone a tracheostomy and then subjected to tracheal intubation. She then developed symptoms of infection. Strain 17ZR101 was isolated from the secretion fluid of tracheal intubation. The patient was treated with meropenem and tigecycline and recovered eventually.

### Murine bacteremia models

*Kp* strain 17ZR101 (serotype K2, ST86), PM45 (serotype K25, ST792) and 17ZR22 (serotype K36, ST437) were grown in LB broth for 18 hours at 37°C. Cultures were then diluted at 1:100 and grown for an additional 2.5 hours to reach the early logarithmic phase. Bacteria were pelleted by centrifugation and washed twice in cold phosphate-buffered saline (PBS) and then resuspended to achieve the desired density. Mice were infected with 1 × 10^4^ colony-forming units (CFU) of 17ZR101 intravenously, 2 × 10^7^ CFU of PM45 or 17ZR22 intraperitoneally. At 12 hpi, the mice organ samples were collected for bacteria burden measurement, metabolic analysis, flow cytometry analysis, and serum was collected for cytokine production analysis.

### Murine pneumonia models

Mice were anesthetized by inhaling isoflurane, and ~10^6^ CFU of the strain resuspended in 20 μL PBS were dripped into the mouse’s nasal cavity. The heads of the mice were hold upright for 30 s afterwards. At 24 hpi, the mice organ samples were collected for bacteria burden measurement, and flow cytometry analysis.

### Single-cell data preprocessing and cell-type determination

Analysis of scRNA-seq data was performed using the package Seurat (version 4.1.1). We retain valid cells based on a number of genes, mitochondrial RNA percentage, and number of UMIs (unique molecular identifiers. The raw counts were then normalized, and highly variable genes were calculated in all datasets with default parameters. We performed Seurat’s standard data integration process based on the identification of anchor cells between the two data sets. After integration, data scaling, PCA, dimensionality reduction (using UMAPs), and clustering were applied for cluster identification and data visualization. Differential expression analysis (DEA) for all clusters was performed to determine their marker genes. Marker genes of all clusters were selected based on a normalized RNA expression value. that being >0.25 log-fold higher than the mean expression value in the other sub-clusters, and with a detectable expression in > 25% of all cells from the corresponding cells.

### Untargeted metabolomics analysis of lung tissues

Lung samples were submitted to BGI Company Limited (Shenzhen, China) for untargeted liquid chromatography-mass spectrometry analysis according to the standard workflow commercially offered by BGI. In brief, metabolites were extracted by methanol: acetonitrile: water (2:2:1, v/v/v). The instrumental setup was based on 2D ultra-performance liquid chromatography (Waters, Milford, MA, USA) coupled to a tandem Q Exactive HF high-resolution mass spectrometer (Thermo Fisher Scientific, Waltham, MA, USA) for separation and detection of metabolites. The chromatographic separation was performed on an ACQUITY UPLC BEH C18 column (Waters) and the column temperature was maintained at 45 °C. The mobile phase consisted of 0.1% formic acid (A) and acetonitrile (B) in the positive mode and 10 mM ammonium formate (A) and acetonitrile (B) in the negative mode. The gradient conditions were as follows: 0–1 min, 2% B; 1–9 min, 2–98% B; 9–12 min, 98% B; 12–12.1 min, 98% B to 2% B; and 12.1–15 min, 2% B. The flow rate was 0.35 mL/min and the injection volume was 5 μL. The mass spectrometry was performed in both positive and negative ionization modes with a spray voltage of 3.8 kV and − 3.2 kV, respectively. The full scan range was 70–1050 m/z. The quality control sample was prepared by pooling equal volumes of each sample and was interspersed for every 10 samples. Data processing was performed using The Compound Discoverer 3.1 (Thermo Fisher Scientific) software, and metabolite identification was conducted by using BGI’s in-house database (BMDB) and public databases (mzCloud, ChemSpider, HMDB, KEGG, LipidMaps).

### Preparation of cells from lung and spleen

Cells collected from lungs and spleens of the infected animals at 12 hpi were subjected to flow cytometry analysis. Cells from spleens were obtained by mashing the organ through a 70-μm cell strainer and collected in a tube containing RPMI 1640 medium supplemented with 5% fetal bovine serum. To prepare lung cells, lung tissues were excised and incubated in HBSS containing 1 x HEPES and Collagenase type I. The tissue fragments were forced through a 70-μm strainer as described above. Red blood cells were lysed with ACK lysing buffer.

### Flow cytometry

Dead cells were excluded from the analysis by propidium iodide (Sigma-Aldrich Corporation, P4170-1G) or ghost dye violet 510 (Tonbo Biosciences, 13-0870-T100) staining in all flow cytometry experiments. Cell suspensions were washed with FACS staining buffer and incubated at 4 °C for 30 min with the following antibodies: APC anti-CD3 antibody (#100235), BV510 anti-CD8a antibody (#100751), APC-Cy7 anti-CD8a antibody (#100713), BV421 anti-CD4 antibody (#100437), PE anti-CD45 antibody (#103106), FITC anti-CD45 antibody (#157213), BV421 anti-CD11b antibody (#101235), APC-Cy7 anti-CD11b antibody (#101225), PE-Cy7 anti-NK1.1 antibody (#156513), BV510 anti-Ly6C antibody (#128033), BV510 anti-CD44 antibody (#103043), PE-Cy7 anti-CD62L antibody (#104417) and FITC anti-CD279 (PD-1) antibody (#135213) from Biolegend and FITC anti-Gr-1 antibody (#11-5931-82) from eBioscience. For intracellular cytokine measurement, cells were incubated with Cell Activation Cocktail (#423303, BioLegend) at 37 °C in the dark for 4 h. Anti-IFN-γ-FITC antibody (#12-7177-81, eBioscience), anti-IL-17A-PC7 (#25-7177-82, eBioscience) and anti-IL-4-PE (#12-7041-82, eBioscience) was used to determine the intracellular expression of IFN-γ, IL-17A and IL-4.

For accessing the IDO1 expression in MDSC and T cells, total lungs cells were stained with AF647 anti-IDO1 antibody (#654003, Biolegend) after incubated with extracellular markers. For assessing FOXP3 and Ki-67 protein expression by flow cytometry, cells were fixed and permeabilized using FOXP3 staining solutions (#421403, Biolegend) and stained with FITC-anti-Foxp3 mAb (#11-5773-82, eBiosciences) and FITC-anti-Ki-67 mAb (#11-5698-82, eBiosciences) following the manufacturer’s instructions. Flow cytometric analyses were performed using a BD FACSCelesta flow cytometer (BD Bioscience). The acquired data were analyzed by the FlowJo software (Version 10.0.7, Treestar, Palo Alto, CA). The flow cytometry gating strategies were shown in S5–**S**10 Figs.

### Isolation of MDSCs and T cells

MDSCs were purified from 17ZR101-infected lungs cells by EasySep Mouse MDSC (CD11b^+^Gr-1^+^) Isolation Kit according to the manufacturer’s protocol (#19867, Stemcell Technologies). Tregs were isolated with the MojoSort Mouse CD4^+^CD25^+^ Regulatory T Cell Isolation Kit (#480137, Biolegend). For isolation of T cells, splenocytes were resuspended in RPMI-1640 supplemented with 10% heat-inactivated FBS and were isolated with the MojoSort Mouse CD3 T Cell Isolation Kit (#480031, Biolegend).

### Suppression assay

To analyze the proliferation of T cells, purified T cells isolated from naive mouse spleens were resuspended in RPMI-1640 supplemented with 10% heat-inactivated FBS and incubated in the presence of CFSE probe (C34570, Invitrogen). A 96-well plate was added 1 × 10^5^ target cells and incubated with anti-CD3e antibody (145-2C11, 5 µg/mL, 16-0031-82, Invitrogen) and anti-CD28 antibody (37.51, 2.5 µg/mL, 14-0281-82, Invitrogen) or complete media for control well in a humidified environment with 5% CO_2_ at 37 °C. MDSCs isolated from the lungs of infected mice were added to the culture at a ratio of 1:1, 1:2, 1:4, and 1:16 MDSC-to-T cells. Three days later, cells were harvested and labeled with following antibodies: APC-CD3, BV510-CD8a, BV421-CD4, and PI. For L-Kyn function analysis, CD3^+^ T cells were cultured in round bottom 96-well plates, in the presence of anti-CD3e antibody and anti-CD28 antibody with defined L-Kyn concentrations, in a final volume of 200 µL. On day 4, cells were harvested, counted and analyzed by flow cytometry.

### Cell apoptosis assay

Apoptotic cells were detected using flow cytometry after staining with annexin-V-FITC/PI dual stain. After coculture, T lymphocytes were harvested, rinsed twice with PBS, and suspended in 500 μL of binding buffer. The suspended cells were incubated for 15 min at 4°C with 5 μL annexin V-FITC solution and then incubated for another 5 min at 4°C after adding 10 μL of PI solution. For each sample, 10,0000 events were recorded. The amount of late apoptosis was determined as the percentage of annexin-V^+^/PI^+^ cells.

### RNA extraction and real-time quantitative PCR analysis

Total RNA was extracted from lung samples, PMN-MDSCs, T cells, MDSCs and Tregs in TRIzol reagent (#15596026, Thermo Fisher Scientific), followed by chloroform extraction and isopropanol precipitation. The extracted RNA was reverse-transcribed into cDNA by using a SuperScript III First-Strand Synthesis SuperMix kit (#11752050, Thermo Fisher Scientific). Real-time quantitative PCR was performed by using a QuantStudio 7 Pro Real-Time PCR System, following the manufacturer’s instructions. *Ido1* and *Il10* mRNA were analyzed by quantitative PCR by using the following primers: Ido1-F: GCTGTTCCT TACTGCCAACT and Ido1-R: AGCAAAGTG TCCCGTTCT; Il10-F: GTGGAGCAGGT GAAGAGTGA and Il10-R: TCGGAGAGAGGTACAAACGAG. cDNA samples were tested in duplicates, and the relative amount of mRNA in different samples was determined by the comparative threshold cycle (ΔΔCT) method, using the glyceraldehyde-3-phosphate dehydrogenase gene (*Gapdh*) for normalization.

### *In vivo* IDO Inhibition by 1-MT

For 1-MT solubilization, 10 mg mL^-1^ 1-MT was dissolved in 5 g L^-1^ sodium carboxymethyl cellulose (CMC-Na) in PBS. For *in vivo* IDO1 inhibition treatment, 100 mg kg^-1^ 1-D-MT was given orally at one day before 17ZR101-infection. Control mice received the sterilized CMC-Na without 1-MT. The health status and weight of all mice were observed and recorded.

### Measurement of bacterial burden in various organs of the test animals

Ten-fold dilutions of tissue homogenate collected were prepared and spread onto MacConkey agar plates containing 2 μg mL^-1^ cefotaxime to determine the bacterial load in different organs of the infected animals. The number of total bacteria was counted and presented as the number of CFU g^-1^ tissue.

### Cytokine analysis

Mice serum was collected in all experiment unless otherwise indicated. The serum level of IL-2 of the test animals was measured by using the IL-2 Mouse Uncoated ELISA Kit (Thermo fisher scientific, 88-7024-77) according to instructions of the manufacturer.

### Statistical analysis

Statistical analysis of data obtained in this work was performed by means of Graphpad Prism 6.0 (GraphPad Software, La Jolla California USA, www.graphpad.com). Statistical analyses on normally distributed data sets were performed using one-way ANOVA with Tukey’s correction for multiple comparisons. The log-rank test was used for comparing survival rate in animal experiments. *P* values < 0.05 was considered significant. Unless otherwise indicated, the survival curve of mice in animal experiments and results of flow cytometry analysis were representative of at least two independent experiments.

## Supporting information

S1 Fig Lymphocytes decreased in mouse pneumonia model. (a) Kaplan-Meier survival curves of C57BL/6 mice were inoculated with 10^6^ CFU of indicated K2-hv*Kp* (17ZR101) strains in an intranasal route. n = 14/group. (b) C57BL/6 mice was infected with 17ZR101 intranasally and bacterial load in various organs of mice was measured at 24 hpi. n = 7. (c) Representative dot plots showing percentages of CD19^+^, CD4^+^, CD8^+^, NK1.1^+^ cells within CD45^+^ cells. Representative bar diagrams showing the percentage and numbers of T cells, B cells and NK cells in 17ZR101-infected lungs. n = 4. Data was represented as mean ± SEM. *p < 0.05, **p < 0.01. *P* values were derived from the log-rank test (a) and two-way ANOVA with Tukey’s multiple-comparison test (d and e).
(TIF)

S2 Fig Trp metabolism pathway activated in 17ZR101-infected lungs. Mice were infected with 1 × 10^4^ CFU of 17ZR101 intravenously. At 12 hpi, the mice lung samples were collected for RNA-Sequencing (Published data, Accession No.: PRJNA851242) and metabolic analysis (this study). (a) Schematic of Trp metabolism pathway. (b) Heatmap of Trp metabolism enzyme mRNA expression level in sham- and 17ZR101-infected lungs. n = 3. The relative intensity of Trp and Kyn in lungs (c) and serum (d) of sham- and 17ZR101-infected mice. n=6. Mean ± SEM. is represented in the data. NS, not significant, ****p < 0.0001. *P* values were derived from the two-way ANOVA with Tukey’s multiple-comparison test (c and d).
(TIF)

S3 Fig 1-MT administration altered immune cell proportion induced by 17ZR101 infection. C57BL/6 mice was treated with 1-MT and then challenged with 17ZR101 intravenously and spleen samples were collected for flow cytometry analysis. (a) Flow cytometry analysis showing percentages of CD11b^+^Gr-1^+^ cells among total spleen cells. n=6. (b) Representative bar diagrams showing the percentage and numbers of CD11b^+^Gr-1^+^ cells in the spleens of 1-MT treated mice. (c) Representative dot plots showing percentages of M-MDSC and PMN-MDSC within CD11b^+^ spleen cells with or without 1-MT treatment. n=6. (d) Representative bar diagrams showing the percentage and numbers of M-MDSC and PMN-MDSC in the spleens of 1-MT treated mice. n=6. Mean ± SEM. is represented in the data. *p < 0.05, **p < 0.01, ***p < 0.001. *P* values were derived from the unpaired two-tailed Student’s *t*-tests (b) and two-way ANOVA with Tukey’s multiple-comparison test (d).
(TIF)

S4 Fig Ido1 knockout improves mice survival with *Kp* infection. Kaplan-Meier survival curves of *Ido1*^*-/-*^ and wild-type mice were inoculated with 2 × 10^7^ CFU of indicated K25-hv*Kp* (PM45) strains (a) and K36-hv*Kp* (17ZR22) strains (b) intraperitoneally. n = 7. The log-rank test was used for comparing survival rate in animal experiments. **p < 0.01, ***p < 0.001.
(TIF)

S5 Fig Flow cytometry gating strategy for MDSC. (TIF)

S6 Fig Flow cytometry gating strategy for M-MDSC and G-MDSC. (TIF)

S7 Fig Flow cytometry gating strategy for CD3^+^Ki-67^+^ and CD4^+^Foxp3^+^ cells. (TIF)

S8 Fig Flow cytometry gating strategy for apoptotic cells. (TIF)

S9 Fig Flow cytometry gating strategy for Th1 and Th17 cells. (TIF)

S10 Fig Flow cytometry gating strategy for CD4 sub-populations. (TIF)

S1 Table Source data for Fig 1. (XLSX)

S2 Table Source data for Fig 2. (XLSX)

S3 Table Source data for Fig 3. (XLSX)

S4 Table Source data for Fig 4. (XLSX)

S5 Table Source data for Fig 5. (XLSX)

S6 Table Source data for lung metabolomics. (XLSX)
