# Supplementary figures and images for: Myeloid-derived suppressor cell inhibits T-cell-based defense against Klebsiella pneumoniae infection via IDO1 production
Source: PLoS Pathog. 2025 Mar 17;21(3):e1012979. doi: 10.1371/journal.ppat.1012979 (PMC11957394; doi:10.1371/journal.ppat.1012979)

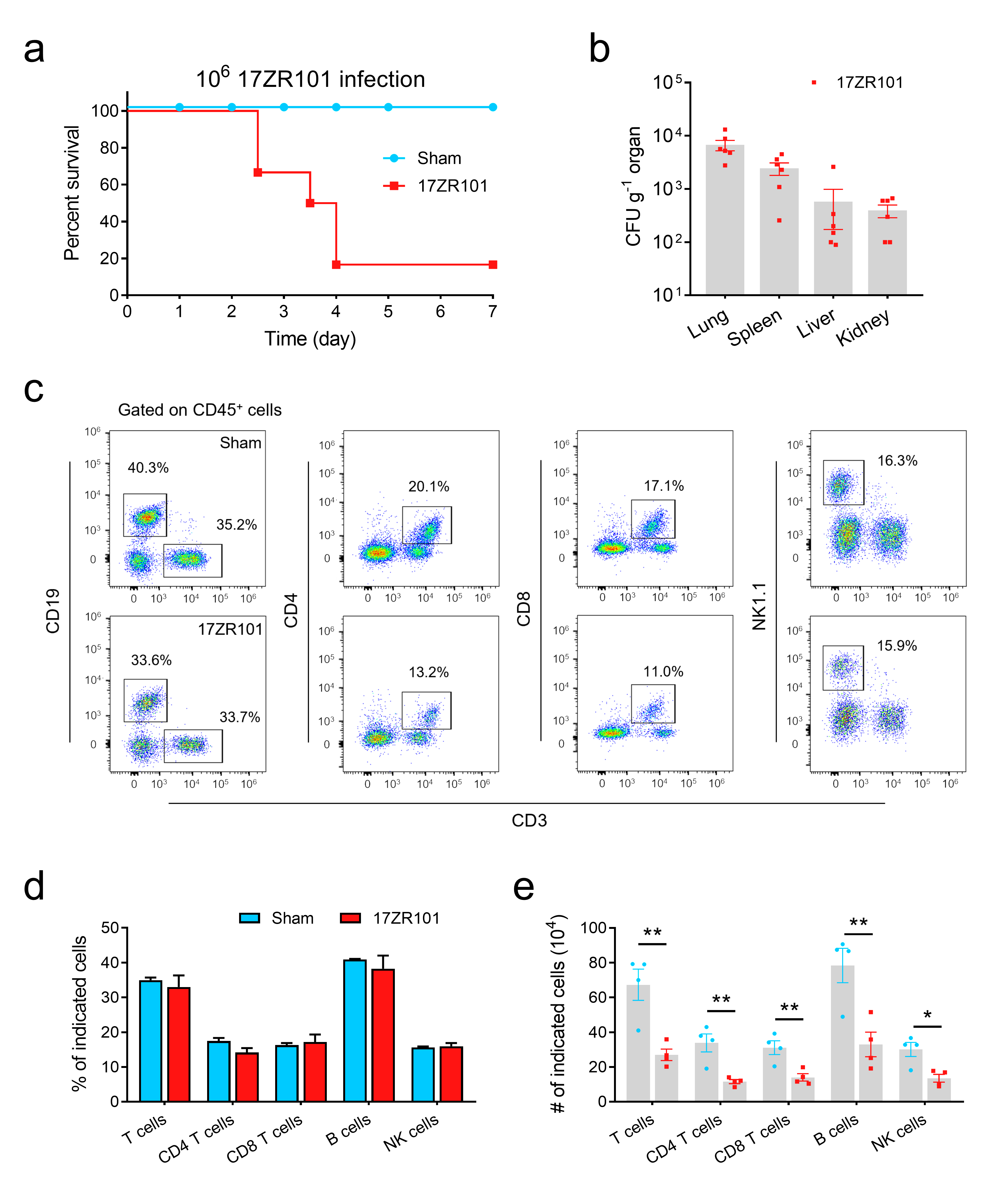

Supplement: S1 Fig — (a) Kaplan-Meier survival curves of C57BL/6 mice were inoculated with 106 CFU of indicated K2-hvKp (17ZR101) strains in an intranasal route. n = 14/group. (b) C57BL/6 mice was infected with 17ZR101 intranasally and bacterial load in various organs of mice was measured at 24 hpi. n = 7. (c) Representative dot plots showing percentages of CD19+, CD4+, CD8+, NK1.1+ cells within CD45+ cells. Representative bar diagrams showing the percentage and numbers of T cells, B cells and NK cells in 17ZR101-infected lungs. n = 4. Data was represented as mean ± SEM. *p < 0.05, **p < 0.01. P values were derived from the log-rank test (a) and two-way ANOVA with Tukey’s multiple-comparison test (d and e). (TIF) [file ppat.1012979.s001.tif]

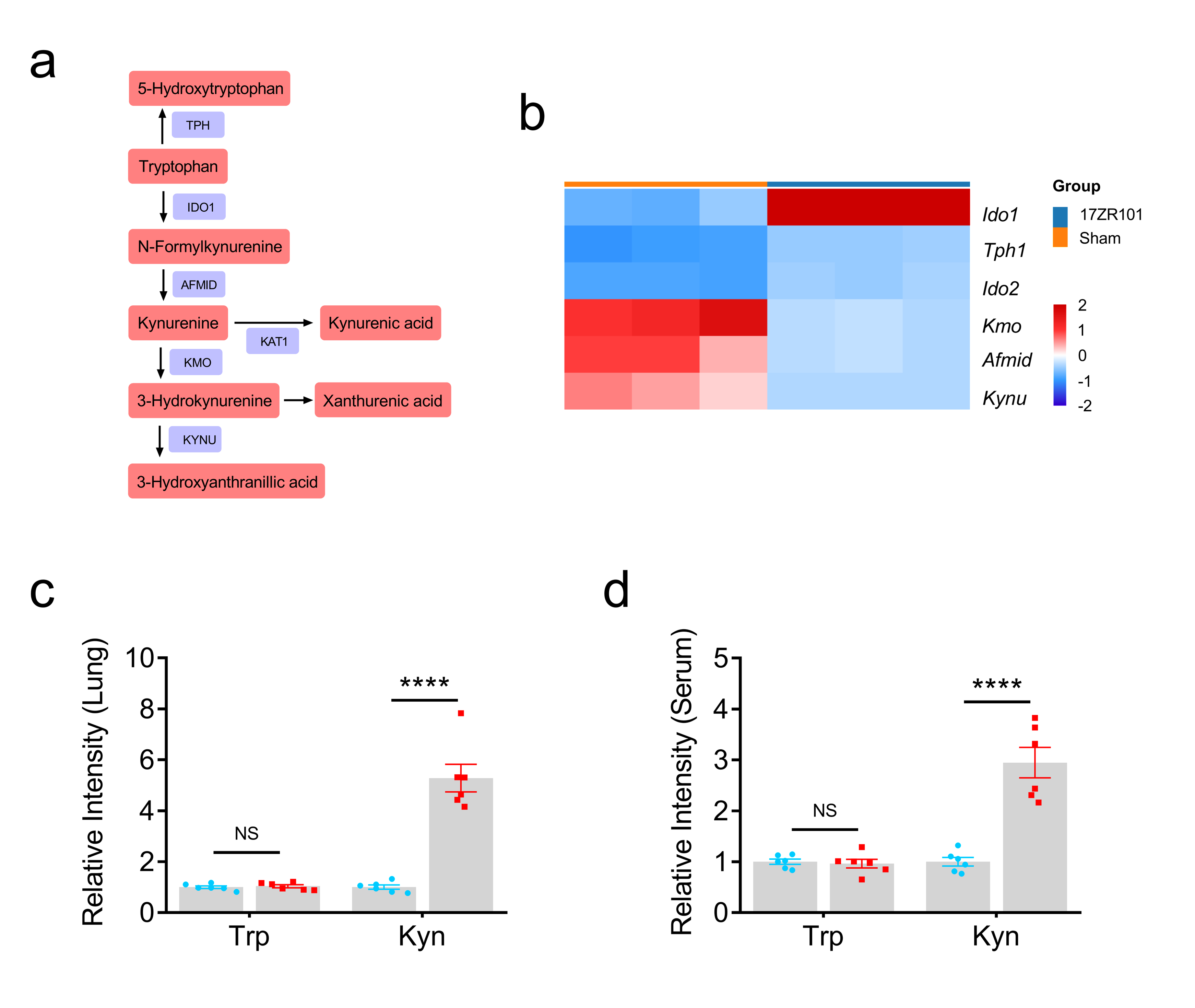

Supplement: S2 Fig — Mice were infected with 1 × 10^4^ CFU of 17ZR101 intravenously. At 12 hpi, the mice lung samples were collected for RNA-Sequencing (Published data, Accession No.: PRJNA851242) and metabolic analysis (this study). (a) Schematic of Trp metabolism pathway. (b) Heatmap of Trp metabolism enzyme mRNA expression level in sham- and 17ZR101-infected lungs. n = 3. The relative intensity of Trp and Kyn in lungs (c) and serum (d) of sham- and 17ZR101-infected mice. n=6. Mean ± SEM. is represented in the data. NS, not significant, ****p < 0.0001. P values were derived from the two-way ANOVA with Tukey’s multiple-comparison test (c and d). (TIF) [file ppat.1012979.s002.tif]

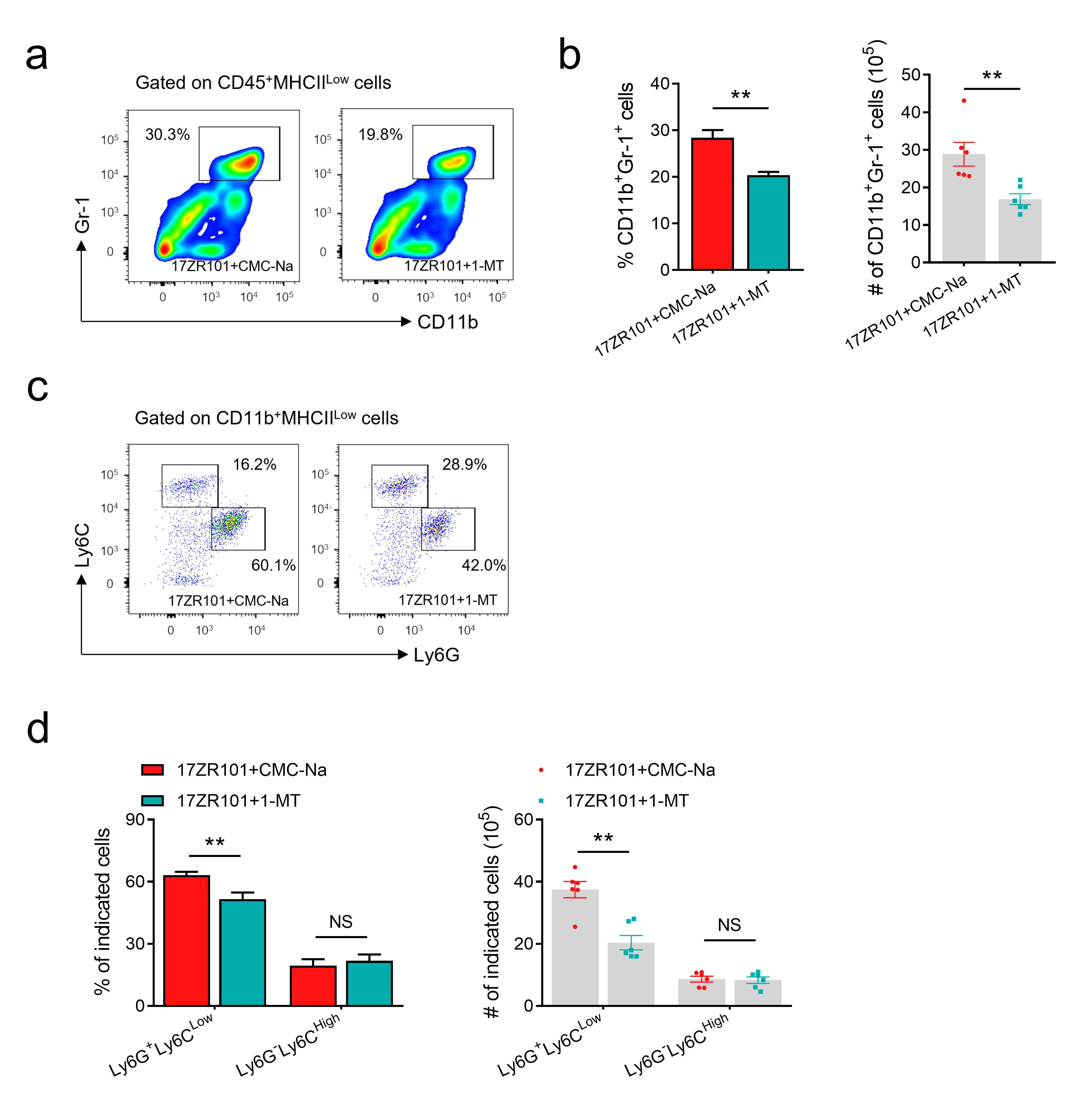

Supplement: S3 Fig — C57BL/6 mice was treated with 1-MT and then challenged with 17ZR101 intravenously and spleen samples were collected for flow cytometry analysis. (a) Flow cytometry analysis showing percentages of CD11b+Gr-1+ cells among total spleen cells. n=6. (b) Representative bar diagrams showing the percentage and numbers of CD11b+Gr-1+ cells in the spleens of 1-MT treated mice. (c) Representative dot plots showing percentages of M-MDSC and PMN-MDSC within CD11b+ spleen cells with or without 1-MT treatment. n=6. (d) Representative bar diagrams showing the percentage and numbers of M-MDSC and PMN-MDSC in the spleens of 1-MT treated mice. n=6. Mean ± SEM. is represented in the data. *p < 0.05, **p < 0.01, ***p < 0.001. P values were derived from the unpaired two-tailed Student’s t-tests (b) and two-way ANOVA with Tukey’s multiple-comparison test (d). (TIF) [file ppat.1012979.s003.tif]

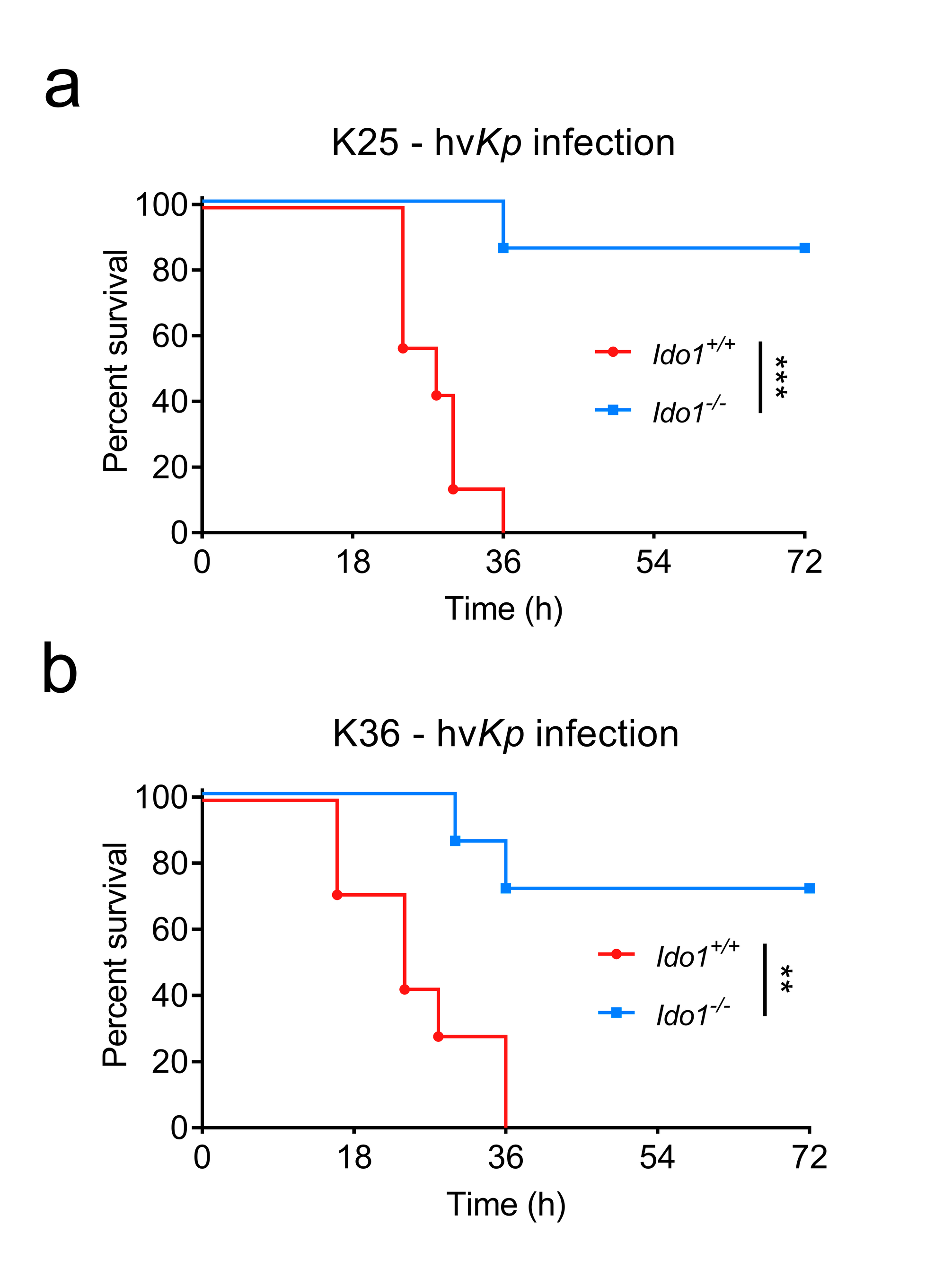

Supplement: S4 Fig — Kaplan-Meier survival curves of Ido1-/- and wild-type mice were inoculated with 2 × 10^7^ CFU of indicated K25-hvKp (PM45) strains (a) and K36-hvKp (17ZR22) strains (b) intraperitoneally. n = 7. The log-rank test was used for comparing survival rate in animal experiments. **p < 0.01, ***p < 0.001. (TIF) [file ppat.1012979.s004.tif]

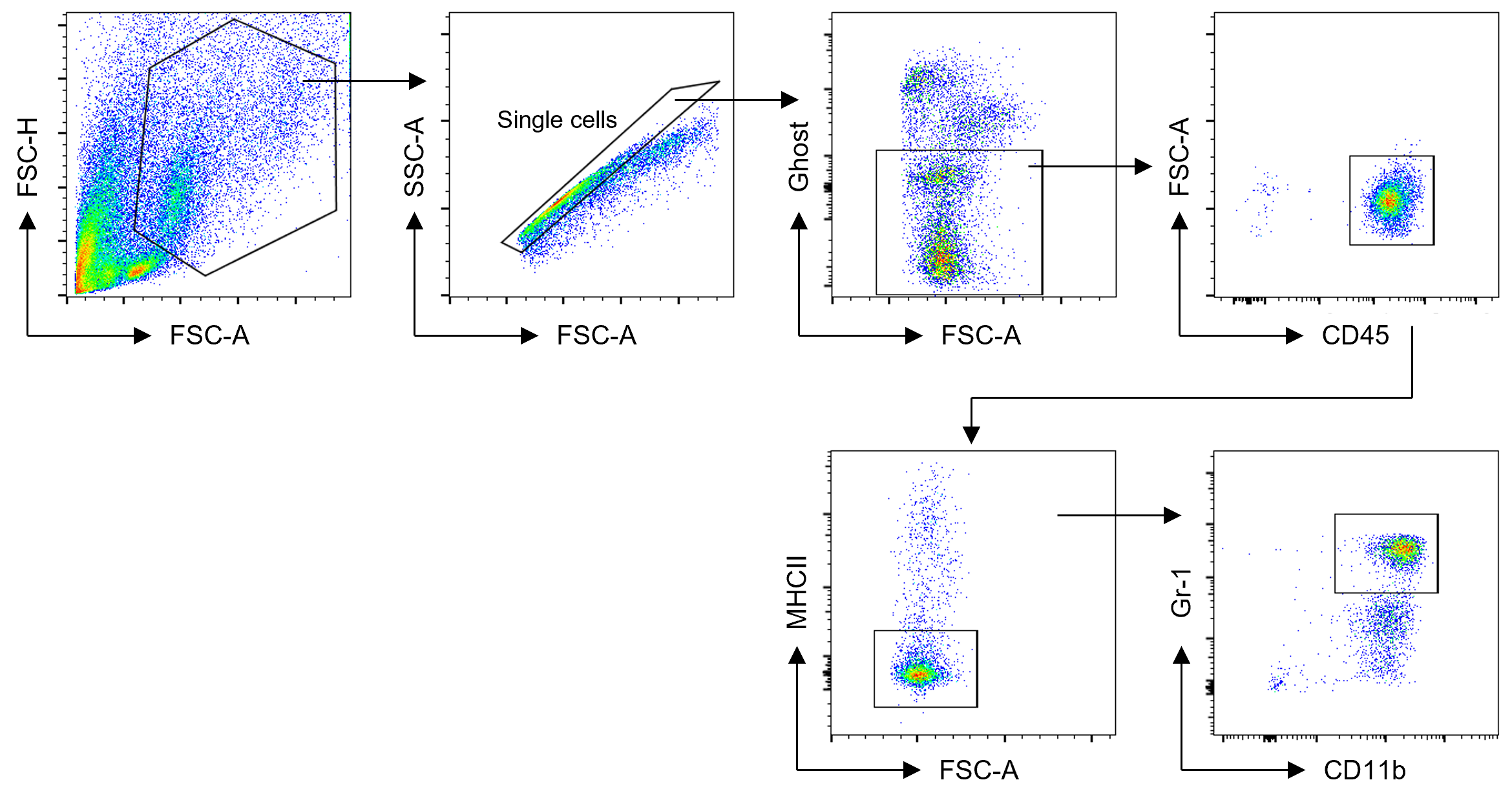

Supplement: S5 Fig — (TIF) [file ppat.1012979.s005.tif]

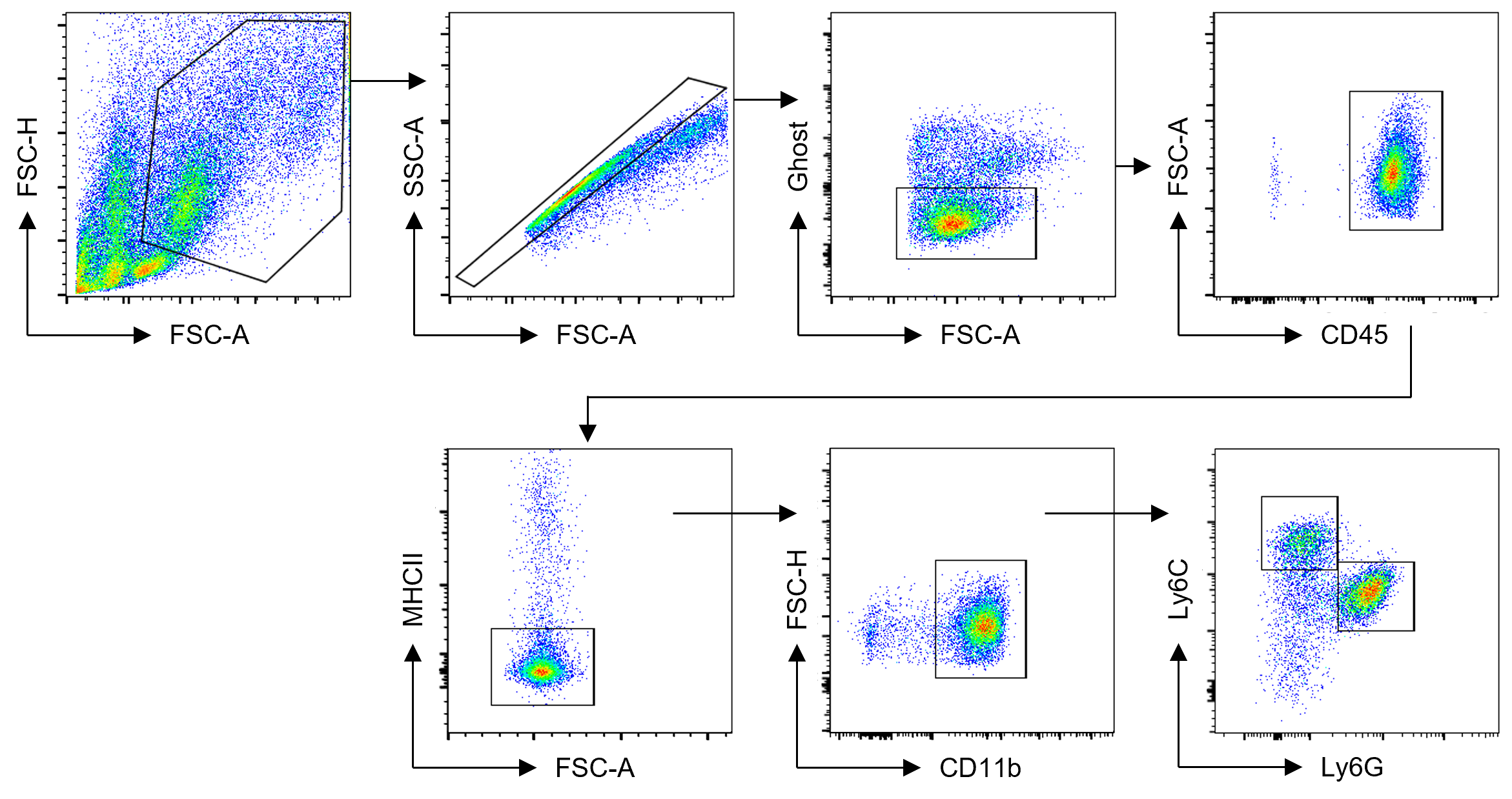

Supplement: S6 Fig — (TIF) [file ppat.1012979.s006.tif]

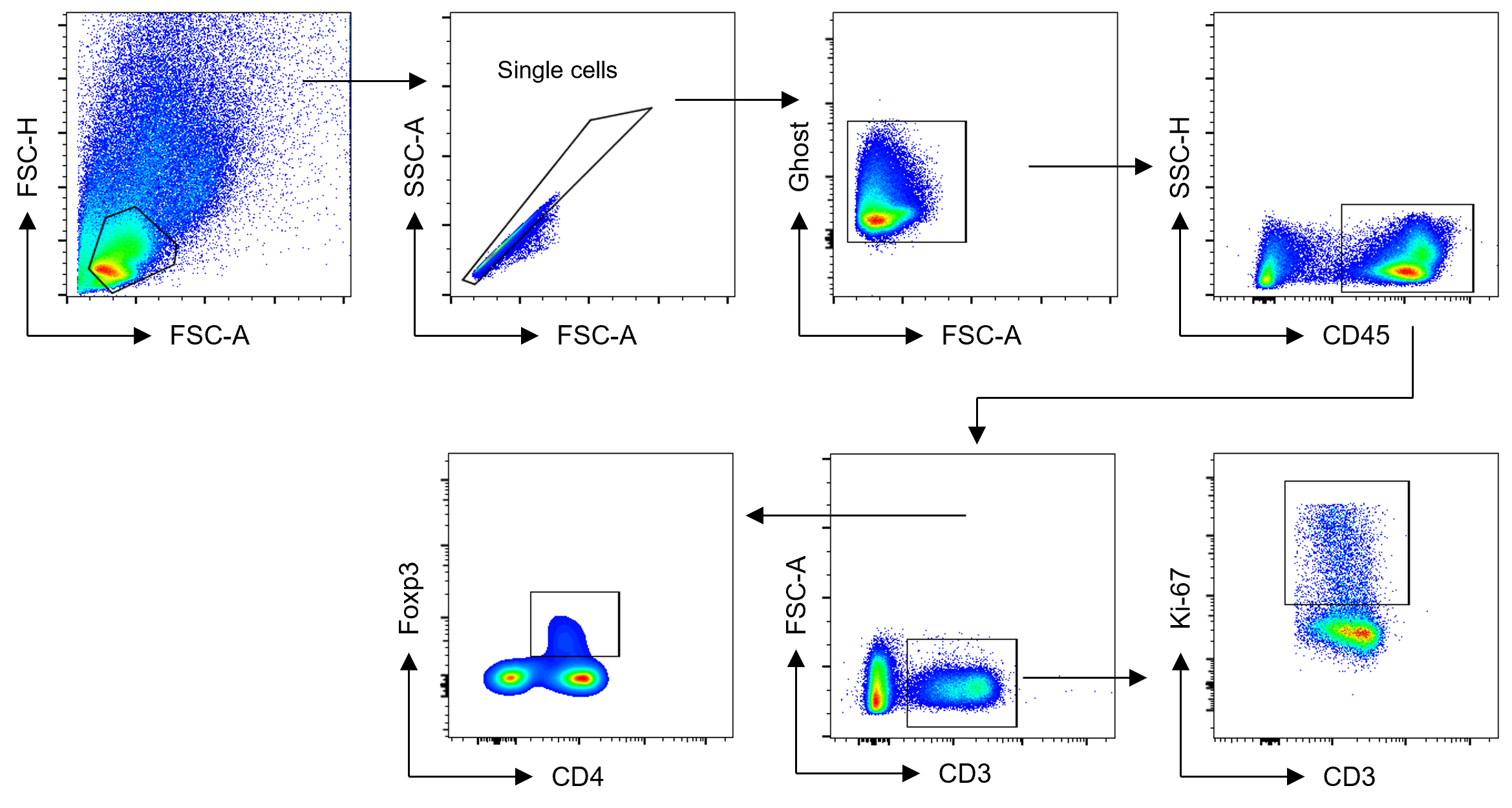

Supplement: S7 Fig — (TIF) [file ppat.1012979.s007.tif]

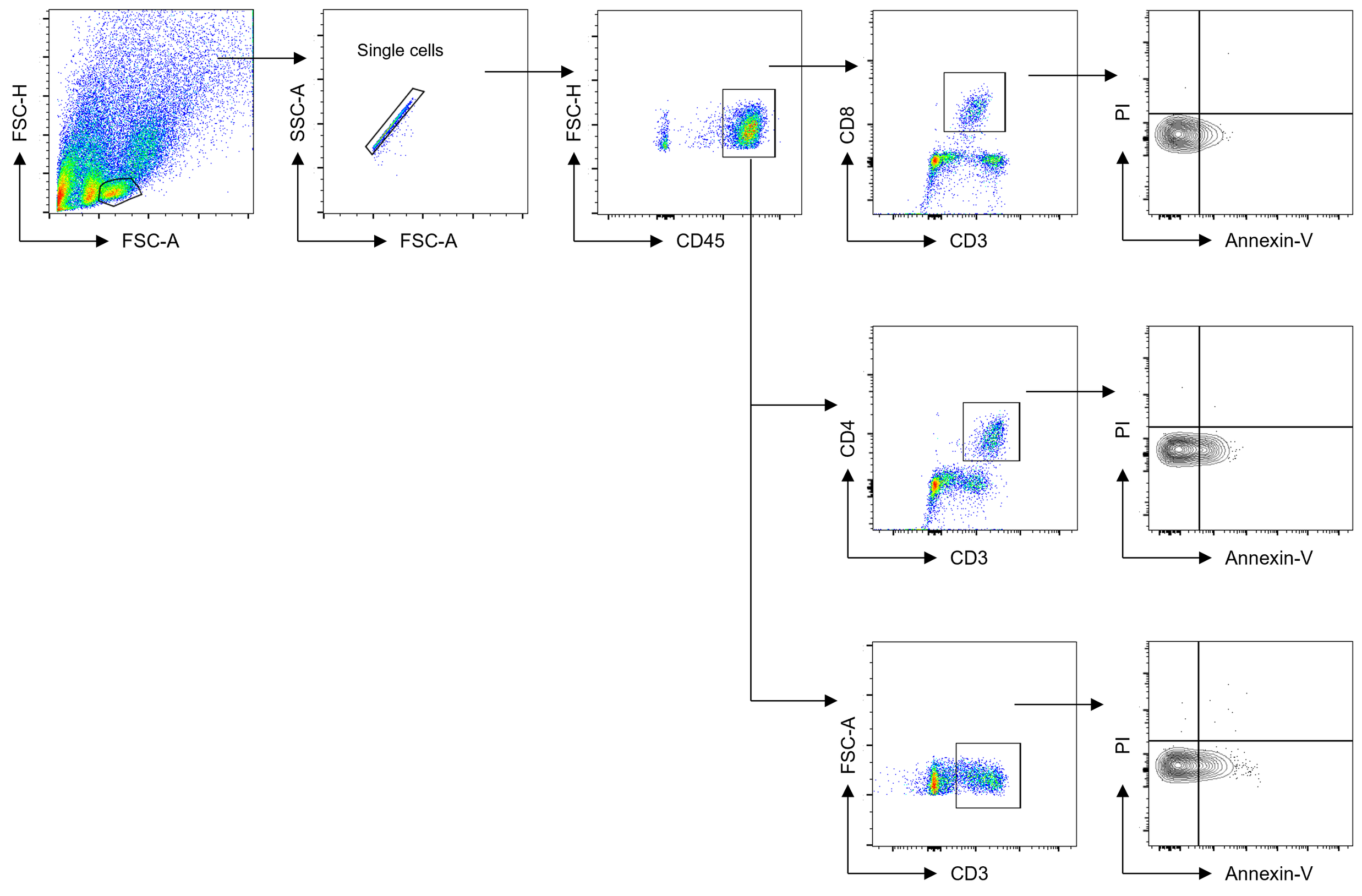

Supplement: S8 Fig — (TIF) [file ppat.1012979.s008.tif]

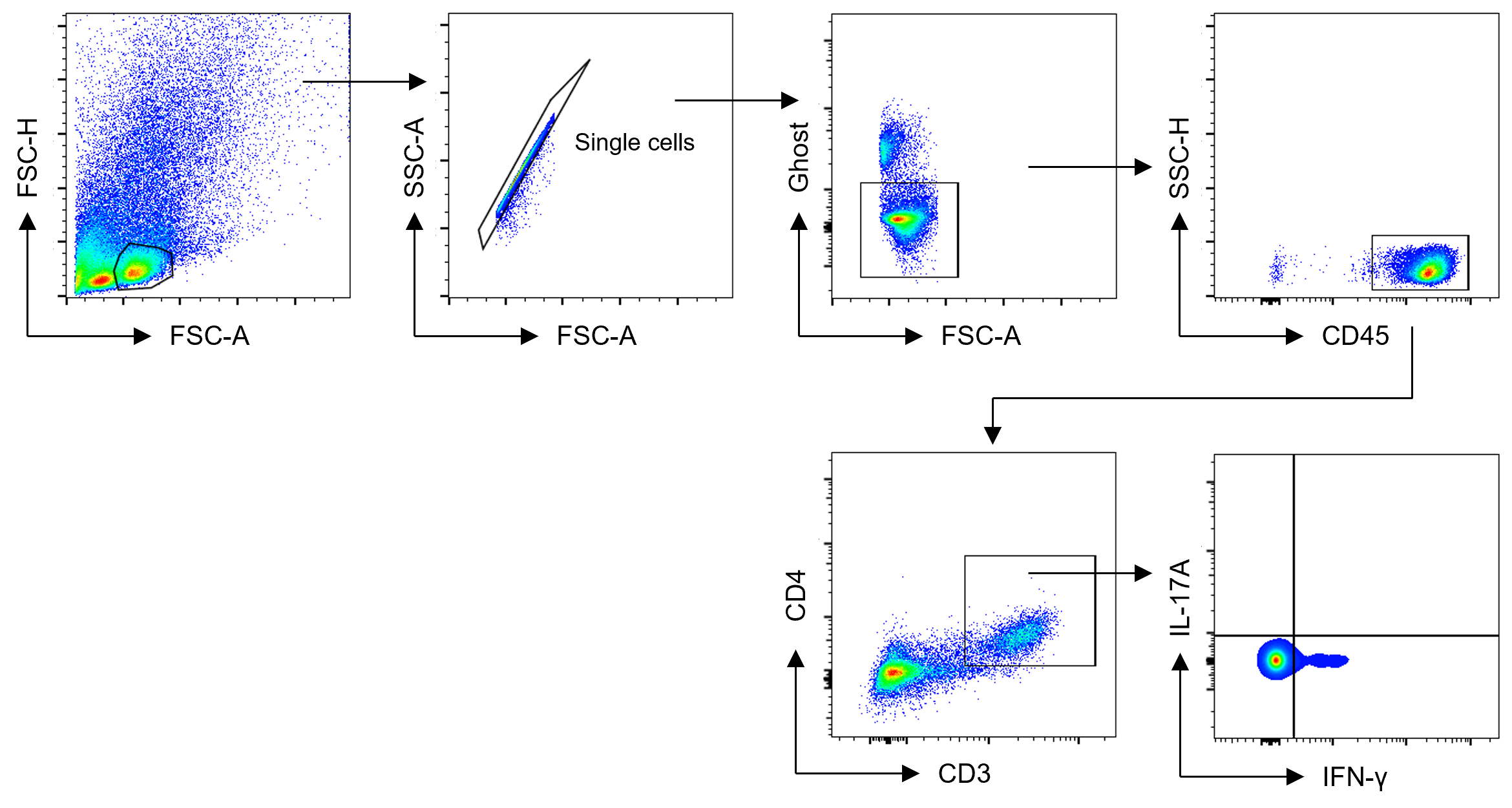

Supplement: S9 Fig — (TIF) [file ppat.1012979.s009.tif]

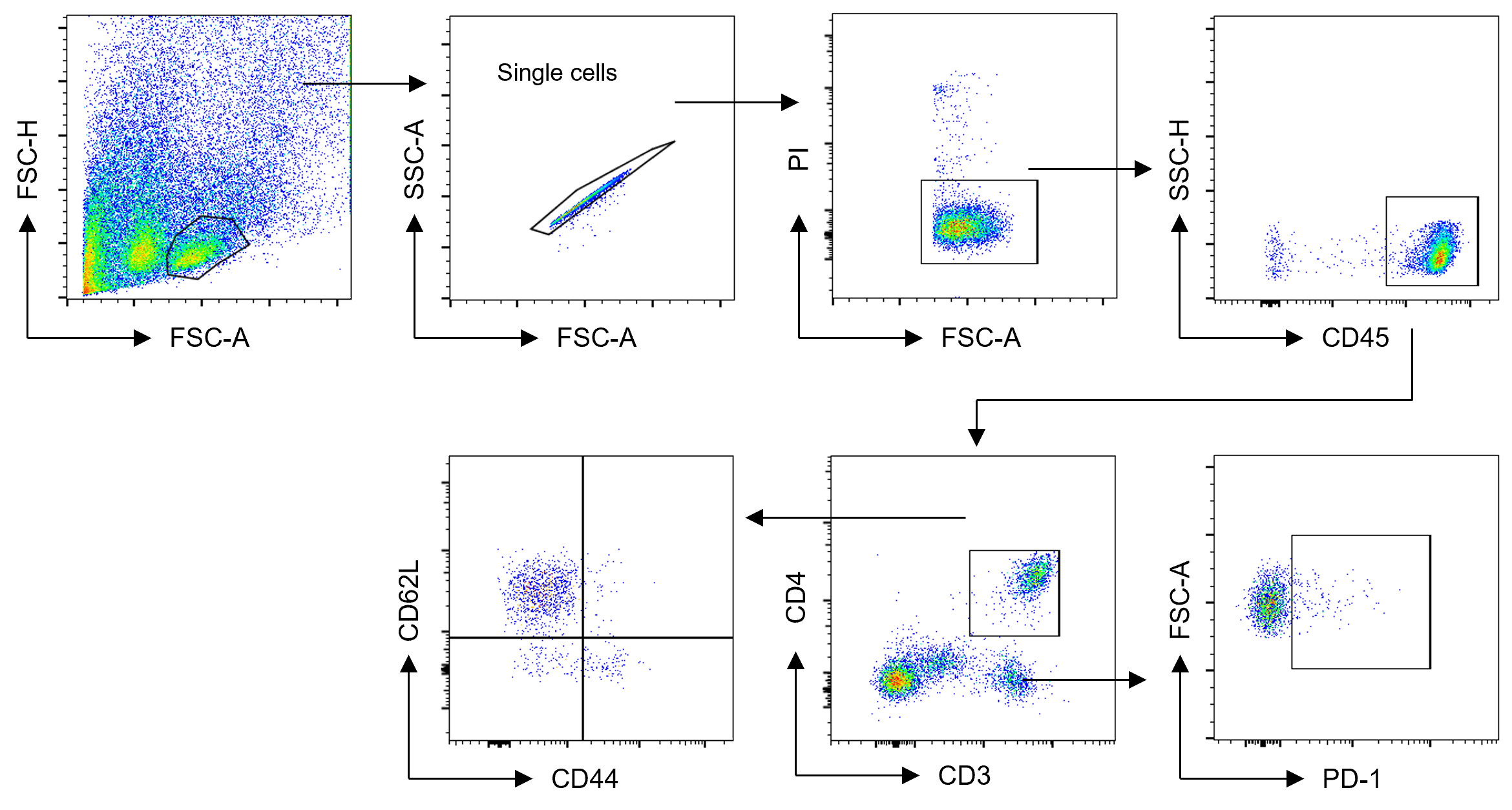

Supplement: S10 Fig — (TIF) [file ppat.1012979.s010.tif]
